# Unravelling the secrets of lesser florican: a study of their home range and habitat use in Gujarat, India

**DOI:** 10.1038/s41598-023-46563-5

**Published:** 2023-11-04

**Authors:** Mohan Ram, Devesh Gadhavi, Aradhana Sahu, Nityanand Srivastava, Tahir Ali Rather, Kapil Bhatia, Lahar Jhala, Yashpal Zala, Indra Gadhvi, Vidhi Modi, Dushyantsinh Jhala, Akshita Patel, Sneha Baraiya, Dhaval Devaliya

**Affiliations:** 1 Wildlife Division, Sasan-Gir, Junagadh, GJ 362 135 India; 2 The Corbett Foundation, Taluka Abdasa, P.O. Tera, Kutch, GJ 370 660 India; 3 Wildlife Circle, Junagadh, GJ 362 001 India; 4 Chief Wildlife Warden, Aranaya Bhavan, Gandhinagar, GJ 382 010 India; 5 Department of Marine Science, M. K. Bhavnagar University, Bhavnagar, GJ 364002 India

**Keywords:** Conservation biology, Grassland ecology

## Abstract

The home range of a species is determined by a complex interplay of extrinsic and intrinsic factors, which can have profound impacts on the species’ resource use. Understanding these dynamics is especially important for conserving critically endangered species. In this study, we used satellite telemetry to investigate the home range of the critically endangered lesser florican (*Sypheotides indicus*) in Gujarat, India. We analysed GPS locations from 10 lesser floricans deployed with GPS/GSM transmitters between 2020 and 2022. The average home range size (95% KDE) was 10.73 ± 10.70 km^2^ (mean ± SD), while the average core area (50% KDE) was 1.95 ± 1.56 km^2^ (mean ± SD). The monthly and daily distances covered were 286.29 ± 599.42 km and 10.11 ± 19.78 km, respectively. Our analysis indicated that suitable habitats and movement patterns were the most important factors explaining the variation in home range size. Specifically, our results suggest that lesser floricans prefer multi-use agro-grassland habitat systems with heterogeneous structures to accommodate different life history requirements. This preference may reflect the depletion and degradation of grasslands across the species’ range. Therefore, managing grassland habitats amidst croplands should be one of the key conservation strategies for the lesser florican.

## Introduction

The home range is the area traversed by an individual in search of necessary resources for survival and reproduction^1–3^. Home range size varies in mammals and birds, depending on factors such as body size and feeding strategy^3,4^. The variation in home range size is influenced by several intrinsic and extrinsic factors specific to each species or population, such as habitat type^5,6^, body weight and size^4^, breeding phase^7^, and population density^8,9^. Similarly, habitat use or selection is also expected to vary based on these factors.

Anthropogenic alteration in the landscape may significantly affect habitat selection and home range patterns in birds. Agricultural areas, which are home to over one-third of all bird species^10^, are of particular interest in understanding their contribution to home range size, especially in birds that inhabit agro-grassland landscapes. Among the factors that determine home range size, food availability is reported to be the dominant and primary factor in birds^11^. For many bird species, agricultural areas provide food resources such as seeds and prey species associated with croplands, which make up a significant fraction of their diet^12,13^.

In this study, we used GPS/GSM transmitters to determine the home ranges, movement patterns, and factors affecting the home range size and habitat use in the critically endangered lesser florican (*Sypheotides indicus*) in and around Blackbuck National Park (BNP) and Kutch Bustard Sanctuary (KBS), Gujarat. This species is endemic to the Indian sub-continent and is the world’s smallest bustard species. Historically, it was widely distributed, and breeding areas occurred in Nashik, Ahmednagar, and Solapur districts of Maharashtra, eastern Haryana, and the Kathiawar Peninsula, Gujarat^14^. However, it now breeds in isolated grassland patches in Gujarat, Rajasthan, Maharashtra, and Madhya Pradesh^15,16^. Habitat loss and seasonal variation in rainfall are the primary factors responsible for its declining population^17,18^. A recent survey^18^ covering most of its known breeding areas in western Madhya Pradesh, southern Rajasthan, and southern and eastern Gujarat and Maharashtra revealed an 80% population decline over the past three to four generations. Lesser florican is a local migrant in India whose movement patterns are linked to seasonal rainfall, and males are reported to show moderate fidelity to their breeding sites^19,20^.

Generally, lesser florican are known to breed in grasslands with sufficient rainfall, ample ground cover, and moderately tall grasses^14,18,21^. They also tend to occur and breed in croplands in areas where grasslands are heavily grazed^22^. Among the croplands, groundnut (*Arachis hypogea*) and soybean (*Glycine max*) are their preferred food sources^23^, while sorghum (*Sorghum vulgare*), rice (*Oryza sativa*), wheat (*Triticum vulgare*), mustard (*Brassica campestris*), maize (*Zea mays*), and cotton (*Gossypium spp*) are visited less frequently^16,17^.

The preferred habitats of lesser florican are fragmented and patchily distributed throughout their range^22^. As a result, these isolated patches serve as important habitats amidst croplands and may even represent the last remaining grasslands available to the species. Therefore, it is crucial to gain a better understanding of the habitat parameters within these fragmented patches that affect the species’ habitat selection.

The size of a bird’s home range varies based on season, breeding status, and available habitats^24–26^. However, telemetry studies that have tagged a relatively small number of birds lack information regarding the variations in the home range of lesser floricans^27–29^. Therefore, we aimed to explain such variation in home ranges of lesser floricans with respect to their habitat and the number of days they were tracked. Our second objective was to investigate the influence of habitat variables on the occurrence of lesser floricans at a fine-spatial scale, and finally, we aimed to provide new insights into their daily movement and migration patterns. Thus, by exploring the interaction of different habitat variables on the home range and habitat use of lesser floricans, we could establish comprehensive space-use patterns to provide information for the future management and conservation of the lesser florican and its habitat.

## Results

### Home range estimation

**Figure 1:**
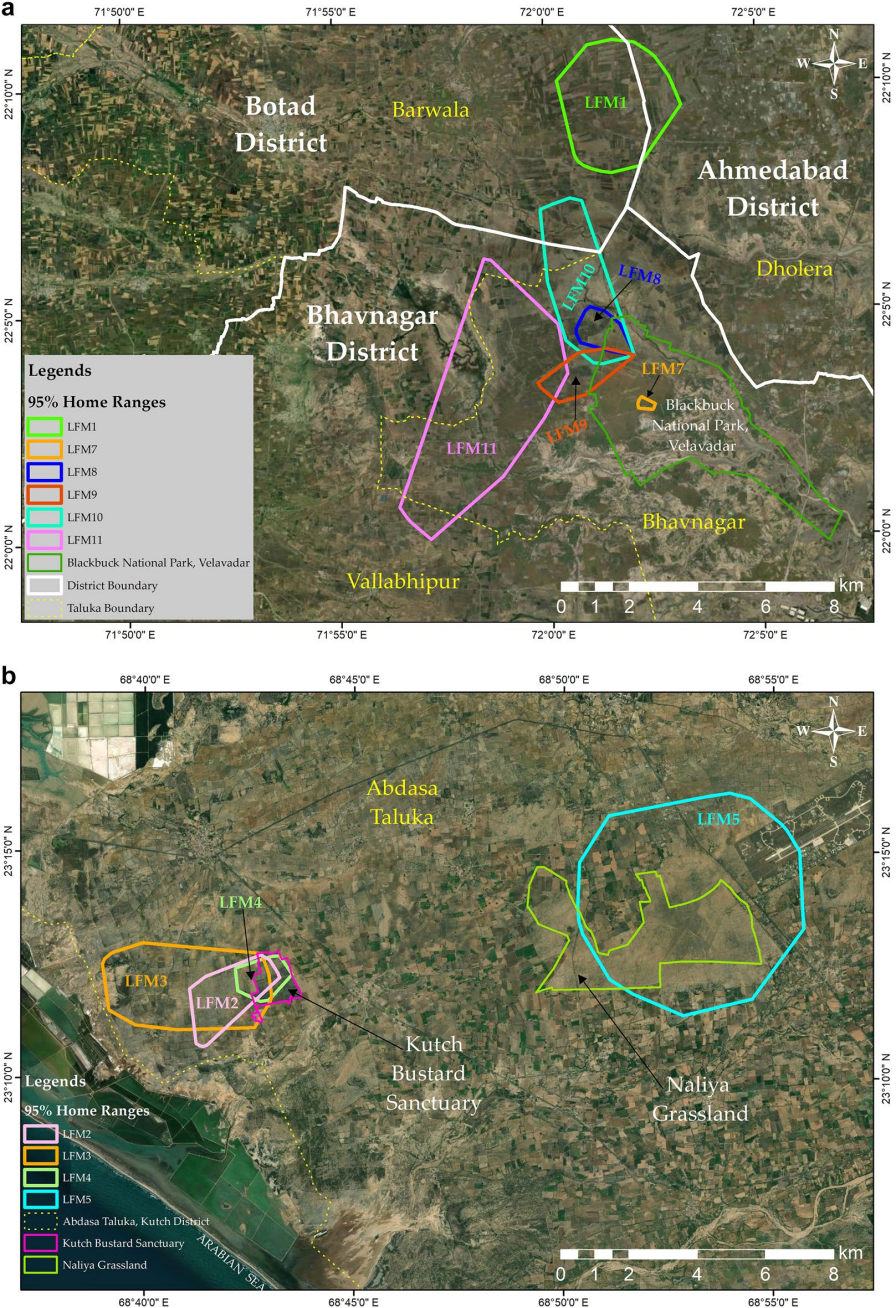
(**a**) Home range polygons of lesser floricans tagged in and around Blackbuck National Park (BNP). Each polygon is colour-coded to represent a different individual. The map was generated using ArcGIS 10.8.1. (**b**) Home range polygons of lesser floricans tagged in and around Kutch Bustard Sanctuary (KBS). Each polygon is color-coded to represent a different individual. The map was generated using ArcGIS 10.8.1.

The average (mean ± SD) home range (95% KDE) and core area (50% KDE) of all tagged birds were estimated to be 10.73 ± 10.70 km^2^ and 1.95 ± 1.56 km^2^, respectively (Table 1). The smallest and largest home ranges were calculated as 0.29 km^2^ and 33.72 km^2^, respectively (Table 1). Except LFM1 and LFM5, all birds showed overlapping home ranges (Fig. 1a,b). The model incorporating individual ID, distance travelled (monthly and daily), tracking time, area of croplands and scrublands (open and dense), and area of grasslands explained about 98% of the variation in home range size (Table 2). Our findings suggest that the presence of suitable habitats and daily life processes such as movement are the best indicators of home range size in lesser floricans. Tracking time accounted for only 24% of the variation in home range size. The average tracking time for all birds was 124.30 ± 106.65 days. Among all the birds, only one bird (LFM5) that was tracked for more than average number of days had a larger home range than the mean home range size. While as only one bird (LFM3) had a smaller home range size than the mean for which the number of tracking days were greater, than the average number of tracking days (Table 1). Open scrub habitat was the only habitat type that could significantly explain the variation (24%) in the home range size of lesser floricans.

**Table 1:** Mean home range (95% KDE), core area (50% KDE), and maximum area used (95% MCP) of lesser florican in Gujarat, India, as calculated using the R package “adehabitatHR”.

ID	95% KDE	50% KDE	95% MCP	Tracking days	No. of locations used
LFM1	18.17	3.64	19.47	90	1046
LFM2	3.69	0.86	4.15	41	2547
LFM3	8.49	1.92	10.70	298	17,139
LFM4	1.49	1.23	6.78	91	5198
LFM5	19.55	1.95	33.65	341	10,809
LFM6*	–	–	–	–	–
LFM7	0.29	0.05	0.24	27	732
LFM8	2.76	0.45	3.07	56	1487
LFM9	4.41	0.95	6.62	103	901
LFM10	14.77	4.13	23.90	103	1060
LFM11	33.72	4.35	39.33	93	3634
Average	10.73	1.95	14.79	124.30	4455.30

The estimates are presented in square kilometres (km^2^).

*Only 277 locations encompassing three days were available; thus, no home range estimates were calculated for LFM6.

**Table 2:** Candidate linear mixed models analysing the factors affecting the variation in home range size of lesser florican (n = 10).

Models	Adjusted R^2^	AIC	ΔAIC	P-value
IDMale + dist_month + dist_daily + tracking_time + grassland + dense_scrub + open_scrub + crop_land	0.98	44.81	0	0.01*
dist_month + dist_daily + tracking_time + grassland + dense_scrub + open_scrub + crop_land	0.90	66.32	21.51	0.02*
open_scrub	0.29	88.67	43.86	0.04*
tracking_time	0.24	89.50	44.69	0.07
tracking_time + dist_daily	0.23	90.32	45.51	0.14
crop_land	0.15	90.71	45.90	0.12
tracking_time + dist_month	0.20	90.80	45.99	0.16
Grassland	0.07	91.64	46.83	0.20
tracking_time + dist_daily + dist_month	0.16	91.76	46.95	0.25
dist_month + dist_daily	− 0.18	93.20	48.39	0.80
Grassland + dense_scrub + open_scrub + crop_land	0.006	93.33	48.52	0.40
dist_month	0.07	93.35	48.54	0.59
dense_scrub	− 0.10	93.69	48.88	0.90

The models are ranked from best to worst according to AIC, a measure of the model’s goodness of fit.

*Indicates the significant models.

We analyzed the effect of daily distance (dist_daily), monthly distance (dist_month), tracking time in number of days (tracking_time), area of open scrub (open_scrub), dense scrub (dense_scrub), area of croplands (crop_land), and area of grasslands within each home range polygon and individual florican (IDMale). Summary statistics include the coefficient of determination (Adjusted R^2^), Akaike Information Criterion (AIC), the difference in Akaike Information Criterion (ΔAIC), and significance values (P-value).

### Habitat selection

The best model for predicting the occurrence of lesser florican included all habitat variables, namely open and dense scrub, human settlement, agriculture, grasslands, and night light pollution (Tables 3, 4). Agricultural fields showed a negative association with predicted occurrence, while grasslands showed a positive association. Lesser floricans selected sites that were significantly different from randomly chosen pseudo-absence locations (Supplementary Information S1). The predicted occurrence was negatively associated with open and dense scrub habitats (Supplementary Information S1). Lesser floricans exhibited a subtle and rather a fragile relationship for habitats with a very small percentage of human settlement (Supplementary Information S1). Our findings also suggest that lesser floricans tended to occur at sites having either a low percentage of night light intensity or higher night light intensity (Supplementary Information S1). This may have been due to the presence of an airfield, which is about 14 km away from Kutch Bustard Sanctuary and less than 1 km from the Naliya grasslands.

**Table 3:** Estimates of fine scale habitat selection of lesser florican using generalised linear models (GLM).

Models	Deviance	AIC	ΔAIC	P-value
Agriculture + grassland + open_scrub + dense_scrub + settlement + night_light	458.13	472.13	0	0.0001***
Agriculture + grassland + open_scrub + dense_scrub + settlement	928.88	940.88	468.75	0.001**
Agriculture + grassland + open_scrub + dense_scrub	929.4	939.4	467.27	0.001**
Agriculture + grassland + open_scrub	992.0	1000	527.87	0.004*
Agriculture + grassland	1001.0	1007	534.87	0.067
Grassland	1001.2	1009	536.87	0.18

The models are ranked from best to worst according to AIC, a measure of the model’s goodness of fit.

The models were fitted using the forward selection process by adding one variable at a time. The variables included grassland (grassland), open scrub habitat (open_scrub), dense scrub habitat (dense_scrub), human habitation (settlement), agriculture (agriculture), and night light intensity (night_light). The models were fitted using a binomial error structure.

***Denotes P < 0.0001, **P < 0.001, and *P < 0.005.

**Table 4:** Estimates of model intercept along with the respective intercepts for each predictor value and their significance values.

Predictors	Intercepts	Significance
Agriculture	− 19.59	0.0001
Grassland	3.95	0.7
Open scrub	− 51.47	0.0001
Dense scrub	− 591.99	0.0005
Settlement	− 229.95	0.05
Night light intensity	− 9.78	0.0001
Model	32.20	0.0001

The intercepts for each predictor value are from the final GLM model that was considered based on the AIC. The predictors were rescaled into the continuous scale and values ranged in percentage from 0 to 1.

### Movement and migration

Lesser floricans covered a monthly distance of 286.29 ± 599.42 km (mean ± SD) and a daily distance of 10.11 ± 19.79 km (mean ± SD) (Table 5). As expected, lesser floricans covered significantly longer monthly distances during migration from the breeding area (F = 17.48, df = 1, p = 0.001). Expectedly, daily movement patterns also differed significantly during migration, with migrating birds covering more distance per day compared to daily movements in the non-migration period (F = 9.08, df = 1, p = 0.01).

**Table 5:** Summary of the monthly and daily distance (mean ± SD) covered by lesser florican, as calculated using the tracking analyst tool in ArcGIS 10.8.1.

ID	Average		Monthly		Daily	
	Monthly	Daily	Minimum	Maximum	Minimum	Maximum
LFM1	213.16 ± 47.00	7.18 ± 2.04	179.88	266.94	5.80	9.53
LFM2	292.34 ± 28.87	14.92 ± 6.36	271.92	312.76	10.42	19.42
LFM3	354.64 ± 269.78	13.08 ± 8.50	117.22	1108.04	5.87	36.95
LFM4	70.54 ± 46.93	2.96 ± 1.12	10.96	114.60	1.80	4.02
LFM5	43.92 ± 16.84	1.79 ± 0.60	16.88	78.13	0.80	3.25
LFM7	45.46 ± 37.54	3.29 ± 2.60	18.92	72.01	1.45	5.14
LFM8	69.48 ± 39.36	3.74 ± 0.39	41.01	114.40	3.31	4.10
LFM9	503.52 ± 854.23	18.59 ± 27.47	54.31	2029.60	4.24	67.63
LFM10	1327.65 ± 1389.59	47.77 ± 51.40	87.97	3505.61	8.40	116.85
LFM11	151.22 ± 31.58	4.99 ± 1.12	114.80	171.02	3.70	5.70

The distance estimates are presented in kilometres (km).

**Figure 2:**
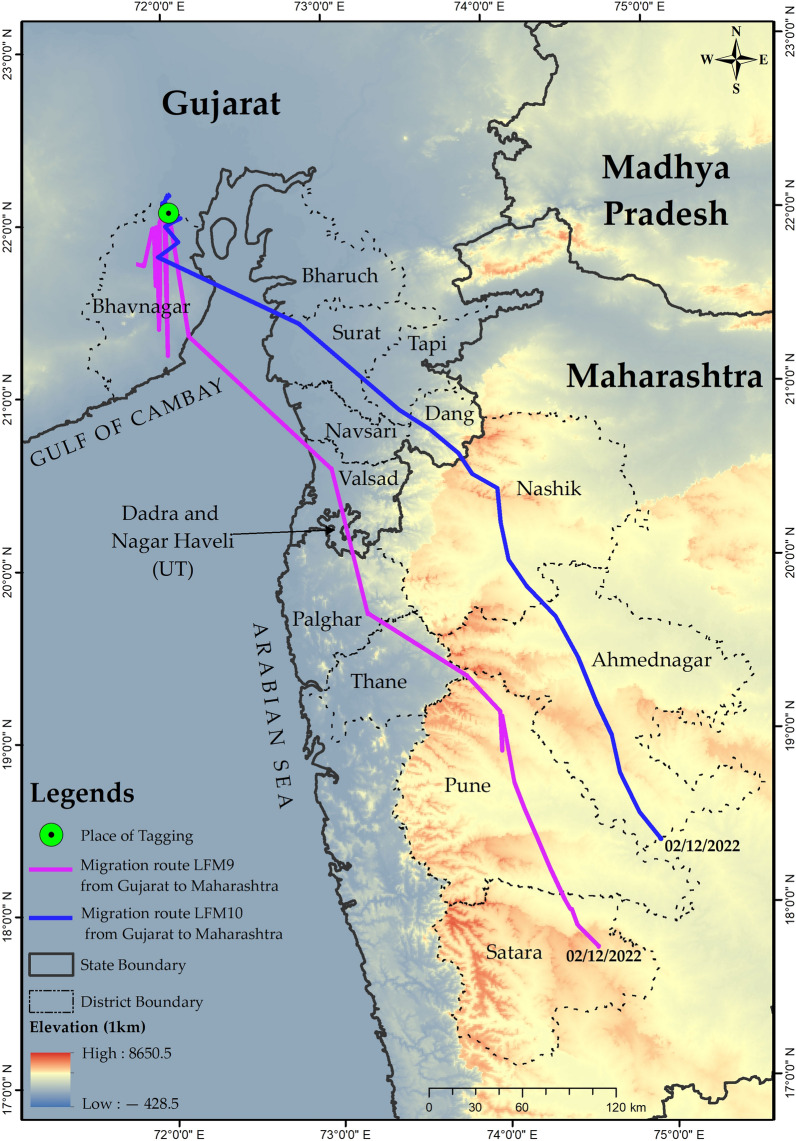
Migration route of lesser floricans from their breeding range in Gujarat towards their non-breeding range in Maharashtra. The migration paths of two individuals, lesser florican-10 (LFM10) and lesser florican-9 (LFM9), are represented by blue and purple lines, respectively. The map was generated using ArcGIS 10.8.1.

Only two of the lesser floricans were found to have migrated from the breeding area. The first signs of migration were observed between October 29th and 30th, 2022, when one of the tagged lesser floricans (LFM9) travelled approximately 25 km southwards from Ratanpur village in the Vallabhipur taluka to Golrama village in the Umrala taluka of Bhavnagar district. It continued its southward journey in shorter distance segments of approximately 20 km until November 11th, 2022, using stopovers in 16 villages distributed in two districts of Gujarat and 15 villages located in three districts of Maharashtra (Supplementary Information S2). LFM9 crossed the Gulf of Cambay (also known as Gulf of Khambhat) between November 18th and 19th, 2022, flying approximately 125 km in one attempt and settling in croplands near Gadaria village of Valsad taluka in Valsad district (Fig. 2). By covering a distance of approximately 100 km in a single day, it crossed Gujarat between November 19th and 20th and entered Maharashtra on November 21st, 2022, where it settled in croplands in proximity to an irrigation canal in Gargaon village of Vada taluka in Palghar district. By December 2nd, 2022, it had travelled approximately 300 km from the previous location in one day and reached Man taluka in the Satara district of Maharashtra (Supplementary Information S2).

During its migration, LFM10 exhibited nomadic behaviour between November 15th and 26th, 2022. It then crossed the Gulf of Cambay on November 26th–27th by flying approximately 95 km and entered Kalwan taluka, located in the Nashik district of Maharashtra, after covering a distance of around 260 km from Gujarat in a single day (Fig. 2). LFM10 used a total of 12 stopovers in four districts of Gujarat and 12 in three districts of Maharashtra during its migration (Supplementary Information S2).

During migration, LFM9 and LFM10 flew at an average elevation of 176.16 ± 225.70 and 313.38 ± 283.90 m (mean ± SD), respectively and covered an average distance of 51.92 ± 12.02 and 116.85 ± 51.20 km per day during migration.

**Figure 3:**
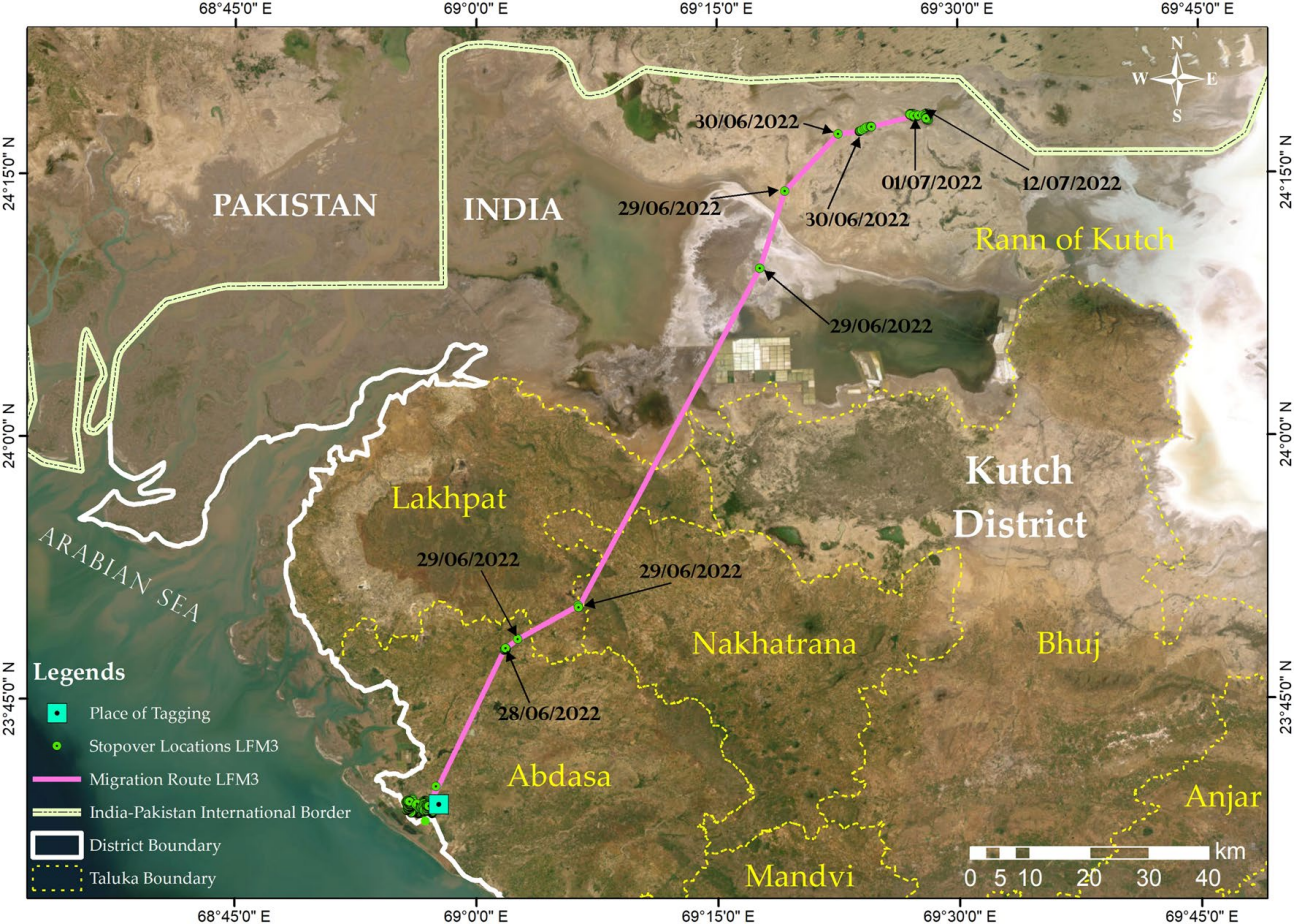
Migration route of lesser florican-3 (LFM3) from Kutch Bustard Sanctuary (KBS) towards a location approximately 5 km of the India–Pakistan International Border in northern Kutch. The map was generated using ArcGIS 10.8.1.

**Figure 4:**
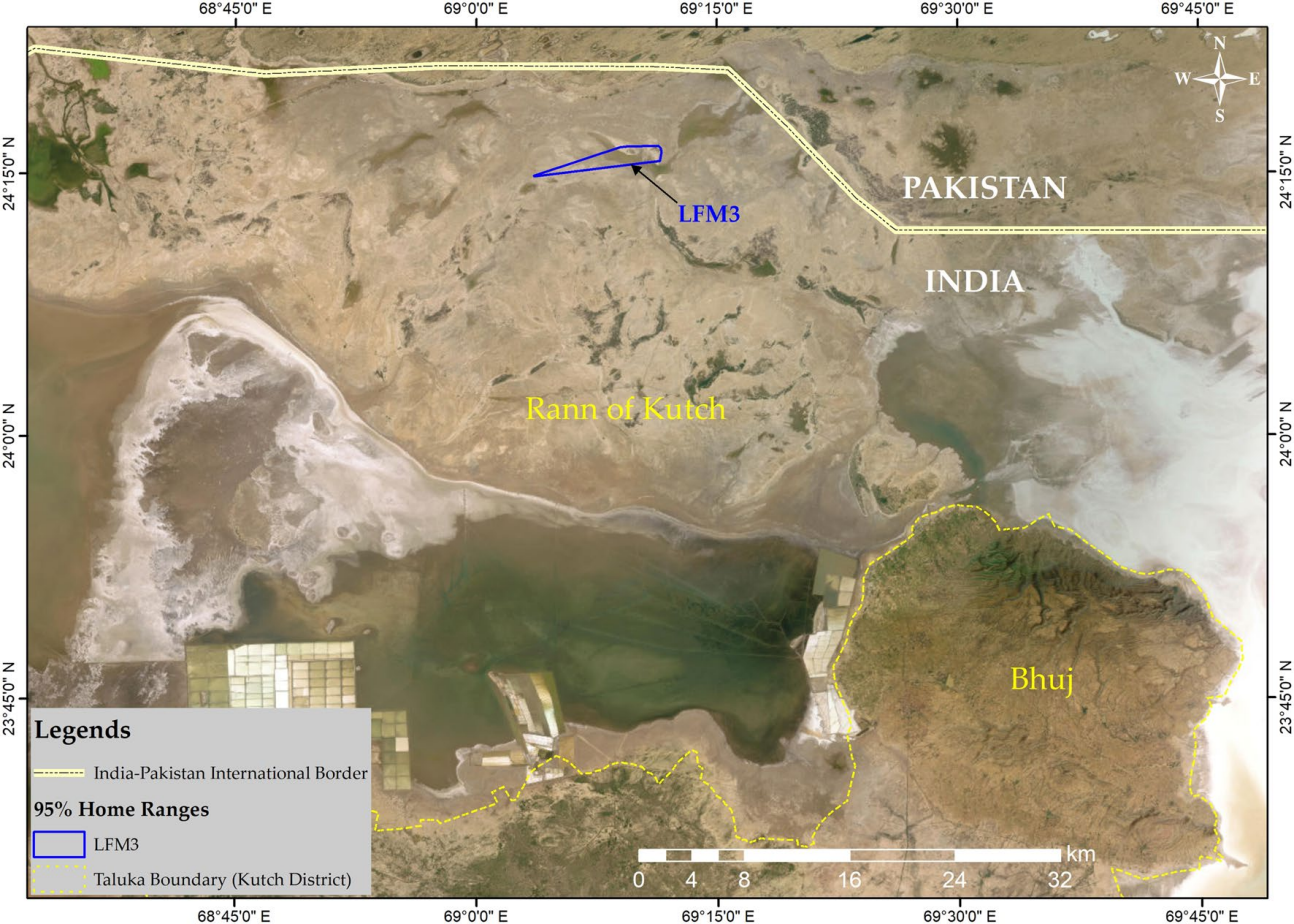
Home range polygon of lesser florican-3 (LFM3) in Greater Rann of Kutch. The LFM3 had dispersed from Kutch Bustard Sanctuary (KBS) to the northern Kutch region near the India–Pakistan International Border. The map was generated using ArcGIS 10.8.1.

The third individual (LFM3) dispersed about 150 km towards the northern Kutch from KBS and settled ~ 5 km south to the India-Pakistan International Border on two small-sized islands (locally known as *beyt*) (Fig. 3). It dispersed on 29th June 2022, making short-duration stopovers near Jagaliya and Dedrani villages in Abdasa and Lakhpat talukas respectively and finally settling in the Greater Rann of Kutch and established a home range of 4.08 km^2^ (Fig. 4).

## Discussion

Both intrinsic and extrinsic factors are reported to influence the home ranges of bustard species^26^. Extrinsic factors such as the availability of grasslands, scrublands, and agriculture fields, as well as movement patterns, were found to have a profound effect and accounted for 98% of the variation in home range size (Table 2). This finding is consistent with a similar study on Canarian houbara bustards (*Chlamydotis undulata fuertaventurae*), which showed that home range size is influenced by season and reproductive status^26^. Although our study focused only on male birds tracked for a single season, the incorporation of individual ID in the model improved the explained variance from 90 to 98% (Table 2), highlighting the importance of intrinsic factors in home range variation. We were limited in our choice of using more intrinsic factors due to the logistic strains. Previous research has suggested that gender and season also play a role in home range size variation in lesser floricans^28^. Mixed models are popular in terms of explaining the variation in home ranges^30^; however, they are sensitive to sample sizes and may not always produce the desired outcome with small samples. Thus, the results of this study may be viewed in terms of being applicable to this particular study area having a small sample size. Our results indicate that the proportion of croplands within the home ranges of lesser floricans ranged from 22 to 78%, with an average of 8.39 ± 9.78 km^2^ of agricultural land and only 3.05 ± 5.22 km^2^ of grasslands within each home range (Supplementary Information S3). Furthermore, other studies suggest that where grasslands are overgrazed, lesser floricans tend to occur in cultivated fields, consistent with earlier studies^18,31^. Although the number of tracking days showed only a weak relationship with home range size (Table 2), we recommend that future studies use larger fixes, longer tracking periods, and monitor more individuals to reduce the bias in home range estimation^32–34^.

The lesser florican, known for its exploded lek mating system^18,31^, is expected to exhibit smaller and adjacent home ranges during the breeding season among males. This mating behaviour has also been observed in other species, such as the Bengal florican (*Houbaropsis bengalensis*) and the African houbara (*Chlamydotis undulata*)^35,36^. Our findings align with a previous study^27^ that revealed males have smaller and overlapping home ranges during the breeding season. This behaviour is typical of species with exploded lek mating systems, as reported in the lesser florican^15^. Although four telemetry studies (including this one)^27–29^ have investigated home range dynamics in lesser florican using satellite telemetry, our study provides novel insights into the spatial and movement ecology of this species by tagging a larger number of birds.

BNP and KBS are two small-sized protected areas surrounded by isolated grassland patches that are scattered within croplands^37^. These patches of habitat are crucial for lesser floricans as they provide important refuge and foraging grounds^36^. Our study found that lesser floricans occurred in areas with low (0.40%) and high (0.80%) percentages of croplands, but the relationship was negative, with occurrences declining as the percentage of croplands increased (Supplementary Information S1). This suggests that the birds may be involuntarily selecting croplands due to their higher percentage around the isolated grassland patches^17^. In another study, lesser floricans were found exclusively in traditional croplands in Shokaliya, Rajasthan, due to the degradation of grasslands^18,38^. As grassland habitats continue to disappear and be modified, lesser floricans have found alternative habitats in these cropland-dominated patches^17^. We found a declining association between the birds and night light intensity, where they tended to occur in areas with moderate to high percentages (0.70% to 0.80%) of night light (Supplementary Information S1). This may be due to the presence of an air force station adjacent to the Naliya grasslands near KBS, where birds were observed displaying adjacent to the runway during monsoon season (D. Gadhavi, personal observation, August 2016). This behaviour may also be due to the nomadic nature of lesser floricans towards the end of the breeding season and the onset of migration. Before migration, lesser floricans that were tagged in and around BNP dispersed for several days and settled towards the Gulf of Cambay, where light intensity was relatively high (> 0.80%).

Although lesser floricans have been reported to occasionally occur in scrub habitats dominated by *Zizyphus* spp. outside of the breeding season^31^, our study found that the birds exhibited a negative relationship with open and dense scrub habitats (see Supplementary Information S1). This suggests that they tend to avoid sites with dense vegetation cover. However, we also found that the birds showed some tolerance towards areas with open scrub habitats compared to those with dense scrub habitats. In both cases, occurrences were clustered around areas with a smaller percentage of scrub habitats.

Gray et al.^39^ reported that human disturbance had a negligible effect on the occurrence of the related bustard species, Bengal florican, in Cambodia. Our results are also similar to this finding; however, we observed that lesser floricans tended to select sites with a low percentage of human habitation (0.006% to 0.016%) (Supplementary Information S1). This preference for areas with low human habitation is consistent with previous studies^22,27,37^. These studies have also shown that lesser floricans tend to occur in croplands only when suitable grasslands are not available or when grasslands are degraded, overgrazed, or subjected to unplanned cropping practices.

Dutta and Jhala^40^ found that the preferred breeding sites for lesser floricans in the Kutch landscape were multiple-use grasslands within the agro-grassland landscape. They also inferred that lesser floricans preferred an optimal mix of grassland proportion and ground vegetation height over intensive agricultural fields or high-intensity grazing^40^. These grasslands included isolated and multiple-use grassland patches amidst the croplands. Related bustard species have also been found to prefer low-intensity crop-grass mosaic habitats^39^. However, this behaviour may not be regarded as an adaptation or ecological plasticity but rather as an involuntary response to habitat loss^17^.

The findings of this study indicate that lesser floricans exhibit a preference for multiple-use agro-grassland habitat systems that contain a heterogeneous range of structures which can accommodate their varying life history requirements. This preference for heterogeneous habitat structures is consistent with that observed in other bustard species that exhibit similar habitat preferences^39,41^. These results highlight the importance of maintaining habitat diversity in conservation efforts aimed at preserving the lesser floricans and other bustard species with similar preferences.

The daily movement and dispersal patterns of lesser floricans remain largely unknown, but initial attempts to study their ranging patterns were made by ringing a large sample of 489 males in the erstwhile Bhavnagar state of Gujarat between 1943 and 1949^42^. While there were indications of strong site fidelity among males, with some returning to the same breeding sites for 20–30 years, ringing records showed only moderate levels of site fidelity, with only 18 of the 489 males being recovered and only 10 of these being captured at the ringing site^18^. In a study conducted by Sivakumar et al.^27^, a radio-tagged male lesser florican was found to have travelled 94 km away from its breeding site in Rajasthan. More recent studies using radio and satellite telemetry have provided new insights into the daily movement and dispersal patterns of lesser floricans^27–29^. For instance, a female was found to have dispersed as far as 776 km from its breeding range in Gujarat to the southern state of Telangana^28^. Here, we also report that one of the lesser floricans tagged in and around KBS settled on two small islands known as *Beyt*, located about 5 km south of the India-Pakistan International Border, and established a home range of 4.08 km^2^. Previous records indicate that lesser florican occurred rarely in northern Kutch^43,44^. This finding represents the first authentic record of lesser floricans in the northern Kutch region, including the Kutch Desert Wildlife Sanctuary (KDWS) and Great Rann of Kutch (GRK), and highlights the importance of these areas as suitable habitats for the species. Our study also provides new insights into the daily movement patterns of lesser floricans, revealing increased daily and monthly distances travelled by some individuals in November, which may be related to migration.

Despite recent studies shedding light on the migration patterns of lesser floricans, the factors that prompt migration in certain individuals remain unknown. As observed in our study, migration was characterised by shorter distance segments of 20–25 km towards south Gujarat, with both individuals crossing the Gulf of Cambay in one attempt and covering a distance of 90 and 125 km. After crossing the Gulf of Cambay, both individuals took only one day to cross Gujarat, settling for shorter durations in agricultural fields near water sources along their migration pathway. These daily movement patterns were consistent with the species’ feeding habits, which involved walking for 5 to 10 m and pausing at shorter intervals to scan the area for threats and prey^45^. Lesser floricans spend a substantial amount of time feeding, especially during the breeding season, when lean birds tend to forage throughout the day^18^.

## Conclusion

In conclusion, the study provides valuable insights into the migration and home range of the endangered lesser florican in a changing landscape. The findings highlight the importance of maintaining grassland habitats interspersed with short organic farmlands to accommodate the different life history requirements of the species. The study also emphasises the need for collaborative efforts and scientific management to mitigate threats such as powerlines and fences around the croplands, especially like fishing nets. With the continued decline of grassland habitats and the increasing pressure of anthropogenic activities, it is imperative to implement effective conservation strategies to ensure the survival of this species.

### Limitations

One of the limitations of the scientific study was that only male birds were tagged, and further observations of the tagged birds in the field were missing, which limits the inferences that can be made about important life-history processes. Additionally, the radio transmitters were inactive during the night, and therefore, important information about dispersal patterns and migration stopovers during the night is missing. The generalisation about their movement and migration patterns could not be ascertained based on the small sample size. The monitoring period of the birds was also relatively short, and a larger sample size, longer tracking periods, and more individuals should be monitored to reduce the bias in home range estimation, as recommended by other studies.

## Materials and methods

### Study area

**Figure 5:**
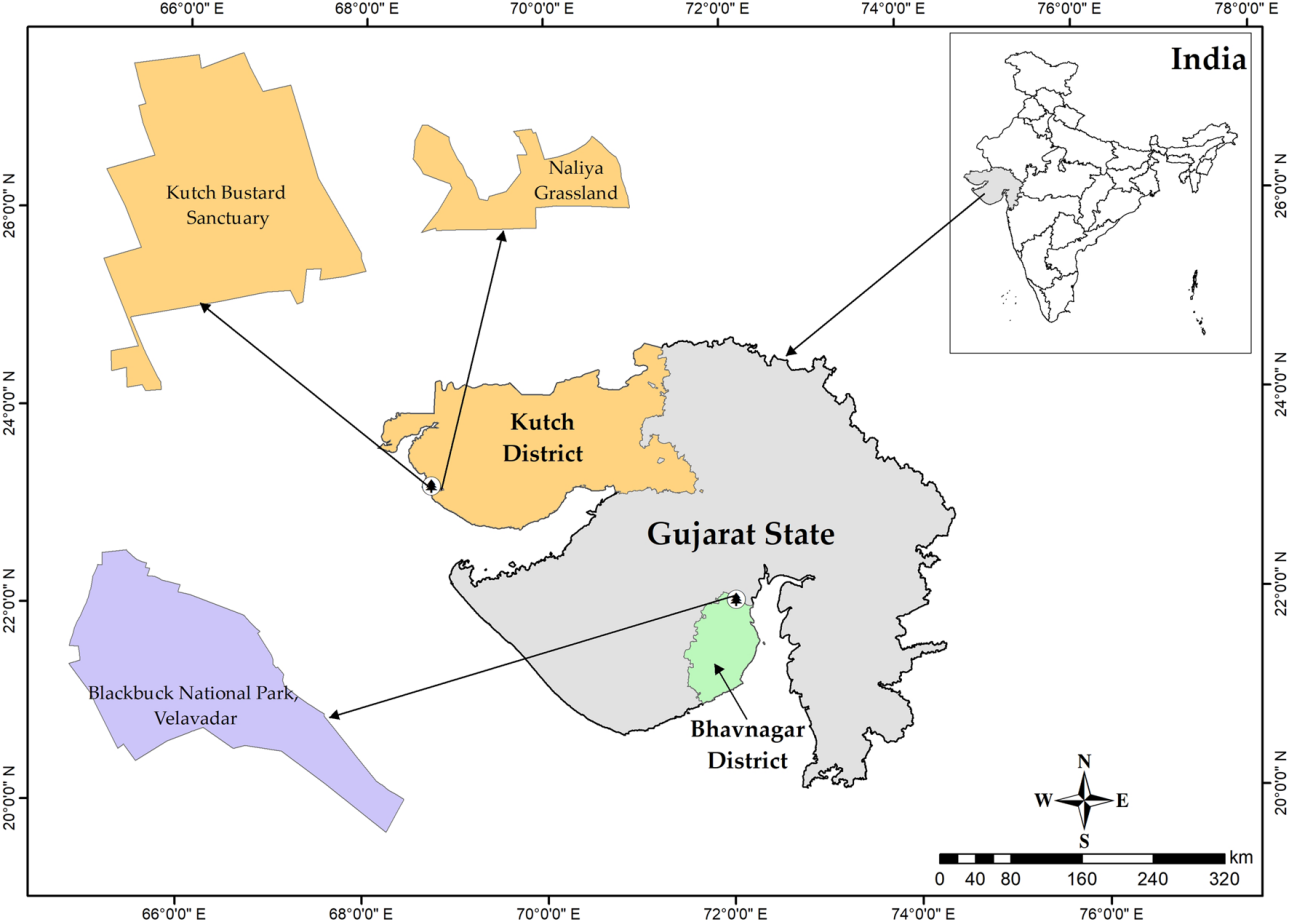
The location of Kutch Bustard Sanctuary (KBS), Naliya Grassland and Blackbuck National Park (BNP). The map was generated using ArcGIS 10.8.1.

Lesser florican individuals were tagged in two distinct sites in Gujarat, India: (1) the Blackbuck National Park (BNP), located in the Bhavnagar district, and (2) the Kutch Bustard Sanctuary (KBS) and Naliya Grasslands, situated in the Abdasa taluka of the Kutch district (Fig. 5). BNP and KBS are separated by an approximate distance of 450 km.

BNP is situated in the semi-arid grasslands and scrubland of the Bhavnagar district in Gujarat, India, covering a total area of 34.08 km^2^ (21° 56′ N, 72° 10′ E) (Fig. 5). It is surrounded by agricultural and low-grazing grasslands in the north and bordered by the Gulf of Cambay in the south, which gets flooded during the monsoon season (July to October). The temperature in BNP varies from 1 to 38 °C in winter (November to February) and 37 °C to 48 °C in summer (March to June). The southwest monsoon (July to September) brings an average of 468 mm of precipitation to the park. BNP is an Important Bird Area (IBA) falling under the semi-arid Gujarat-Rajwada biotic province of the semi-arid bio-geographic zone^46^. The major representative habitat types of the park include grasslands, shrublands, saline land, and tidal mud-flats. Grasslands cover about 60.9% of the park area, while shrublands cover 15%^47,48^. The floral diversity of the park is represented by 39 species of grasses and 46 species of sedges, shrubs, and trees^49^. The dominant grass species in the park include *Dicanthium annulatum*, *Sporobolous virginicus*, *Sporobolous coromandelianus*, and *Sporobolous madernspatensis*^47^. Common shrub and medium-sized tree species found in BNP include *Salvadora, Acacia nilotica*, *Zizyphus*, *Capparis*, and *Suadea*. The park is home to more than 185 species of birds and is the largest roosting ground for the four migratory species of harriers, namely the Western Marsh Harrier (*Circus aeruginosus*), Montagu’s harrier (*Circus pygargus*), Hen Harrier (*Circus cyaneus*), and Pallid harrier (*Circus macrourus*). During the monsoon season, BNP has the highest concentration of lesser florican individuals in India.

KBS, covering an area of 2.08 km^2^, is situated between 23° 11′ N and 68° 44′ E in Abdasa taluka, on the western boundary of the Kutch district (Fig. 5). The Kutch landscape is characterised by flat terrain and Northern Tropical Thorn vegetation^50^, consisting of a blend of grasslands such as *Dicanthium*, *Aristida*, *Cymbopogon*, and *Chrysopogan*, and scrub vegetation, including *Acacia*, *Capparis*, *Zizyphus*, and *Prosopis*. Croplands of groundnut, cotton, and millet are interspersed within the landscape^40^. The average minimum temperature in the area is 5 °C, while temperatures range from 40 to 45 °C during the summer season. The region receives an average annual precipitation of 384 mm^51^. Abdasa taluka is home to over 33% of the total (612) bird species found in Gujarat^52,53^. Naliya grassland is also an Important Bird Area (IBA) site of Kutch, belonging to the Criteria A1 category, and supports a rich diversity of avian fauna, including the critically endangered Great Indian Bustard (*Ardeotis nigriceps*)^49^. Furthermore, it is the sole location in India that harbours three species of bustards: the Great Indian Bustard, Asian houbara (*Chlamydotis macqueenii*), and lesser florican^49^. In the past, approximately 60 displaying male lesser floricans have been observed in this area^16^.

### Bird capturing and transmitter deployment

The capturing and tagging of lesser floricans for satellite telemetry was conducted in two phases. During the first phase in 2020, one male and one female were tagged in BNP^28^. In the second phase (2021–2022), an additional ten male lesser floricans were tagged with satellite transmitters, resulting in a sample of 12 individual birds. The characteristic breeding habit of male performing their courtship display from a specific place in their black breeding plumage makes them conspicuous and easy to detect. While females remain in their usual camouflaged plumage and are also skulker, decreasing their probability of being detected. As trapping a skulker female of a critically endangered species on her nest may disturb her breeding success, we intentionally avoided this to adhere to conservation ethics. Additionally, due to the short breeding season, we had a limited timeframe for trapping these birds. Therefore, only males had the highest chance of being captured. Of these, six were deployed with solar-powered GPS-GSM transmitters in BNP. Out of the four birds tagged in and around KBS, three were deployed with GPS-GSM transmitters while one individual was tagged with the solar-powered PTT satellite transmitters (Supplementary Information S4). The tags retrieved from the dead birds were re-used during the second phase of tagging.

The birds were captured by experienced professional trappers who had prior experience of capturing bustards within the study area^28^. We followed the methods and protocols that were developed in our earlier experience^28^, where a team of researchers and resource persons from the forest department observed the birds’ activities for at least a week before the tagging. Subsequently, traps were laid before dawn at suitable areas where the birds were likely to occur. These sites were selected based on the detailed observations made by the team of observers as described above. A typical noose trap consisted of an anchor line with several monofilament fishing line nooses attached to it. We secured the ends of the anchor line to prevent the trapped bird from flying off with the trap. All necessary precautions were taken to minimise stress to the birds. The capturing team consisted of four to five persons who remained concealed at an approximate distance of 250–300 m from the traps for quick access to them. The captured birds were handled with care, and their heads were covered to minimise stress. Supplementary Information S6 contains photographic details of the tagging process.

All birds were deployed with backpack-mounted solar-powered GPS-GSM satellite transmitters. Two of the transmitters, weighing 12 g each, from Microwave Telemetry utilised the Argos Satellite Data Collection Relay System (CLS America, Lanham, MD, USA), while the remaining eight transmitters, weighing 10 g each, from Ornitela, UAB, Vilnius, Lithuania, used the GSM network (cellular phone) to transmit data. While satellite transmitters offer the advantage of providing near-real-time acquisition of location data from tags placed almost anywhere on the globe, they come with the drawbacks of high cost and substantial battery power consumption^54^. Additionally, their accuracy is sensitive, with reported variations ranging from 100 m to approximately 50 km^54^. In contrast, GSM transmitters store location data and transmit it over the cellular network whenever available, reducing their power requirements. Furthermore, we found that the GSM transmitters are a more economically practical choice compared to satellite transmitters. The weight of all transmitters was less than 3% of the body mass of the tagged lesser floricans (Supplementary Information S4).

In this study, data was analysed for only ten individuals. The female tagged in phase 1 in 2020 died due to a collision with a power line during migration^28^. One individual (LFM2) was caught in a fishnet in an agricultural field and later died due to sustained injuries (Supplementary Information S5). The data was also insufficient for LFM6 to be considered for further analysis (only 3 days of tracking data were available). Data for the male lesser florican tagged in phase 1 (LFM1) was also included from December 2021 to February 2022, which was not previously used in Ram et al.^28^.

### Data collection and processing

A total of 52,889 locations were recorded, and 45,081 of these locations were used for analysis. Inconsistent fixes such as records with missing timestamps, missing coordinates, and low-accuracy class fixes were removed using software like MTI GPS data parser (for microwave telemetry satellite transmitters) and Microsoft Excel (Version 2302 Build 16.0.16130.20298). The transmitters recorded the location of each individual bird every 30 min over a 24-h cycle.

### Home range estimation and variation

We estimated home ranges using kernel density estimators (KDE) and minimum convex polygons (MCP) with the R package “adehabitatHR”^55^ in R^56^. To define the total home range size, we used 95% kernels (95% KDE), and for delimiting the core areas or most intensively used areas, we used 50% kernels (50% KDE). We determined the maximum area used by individuals using a minimum convex polygon at 95%. The smoothing parameter for all home range estimations was set to the reference bandwidth ‘href’^57,58^. To analyse the variation in home range size, we used linear models (LM) with fixed effects. Since none of the tagged birds were female, nor did all tagged birds migrate, we analysed the variation in home range size in relation to the area of each particular habitat type within home range polygons, the number of tracking days for each bird, mean monthly and daily distance covered by each bird. We calculated monthly and daily distances covered using the Tracking Analyst tool in ArcGIS 10.8.1 (Redlands, ESRI, California, USA). The best model was selected based on the lowest Akaike Information Criterion (AIC)^59,60^, and linear models were calculated using the “lme4” package in R^61^.

### Habitat use and selection

To investigate the habitat types preferred by lesser floricans within their home ranges, we used Generalised Linear Models (GLM). GLM provides a simple interpretation of the binary data (lesser florican presence–absence) because the coefficients represent the simple log-odds of the event occurring, which was important in our case as we were interested in determining the influence of individual predictors on the probability of lesser florican occurrence. On the other hand, multinomial models are complex in interpretation as coefficients represent the log-odds of one category relative to a reference category. Our dependent variable was lesser florican presence–absence data, while four habitat categories (open scrub, dense scrub, croplands, and grasslands) and two disturbance factors (night light and human settlement) served as explanatory variables. We specifically considered these habitat variables as they are reported from other studies to be strong indicators of lesser florican presence^18,21,22,31^. Before our analysis, we prepared the data for GLM modelling in the following way. We created a circular buffer of a 200 m radius around each presence location and generated twice the number of pseudo-absence locations using ArcGIS 10.8.1. We were primarily interested in assessing the fine-scale habitat use of lesser floricans and thus chose a 200 m spatial scale corresponding to the fourth-order habitat selection as described by Johnson^62^. The fourth-order habitat selection corresponds to the actual sites of resource utilisation such as food, nests, and shelter within the home ranges of the species^62^. Thus, a 200 m radius buffer provided an appropriate spatial extent to assess the fine-scale habitat use of lesser florican. We ensured that each pseudo-absence location was at least 200 m away from each presence location to avoid pseudo-replication. We also applied a spatial filtering of 200 m to the presence location data to reduce spatial autocorrelation, which is an inherent bias in spatial data due to the non-independency of variables sampled at nearby locations. We tested the random distribution of presence locations using Global Moran’s I^63^ in ArcGIS 10.8.1 and retained an equal subset of pseudo-absence locations to counteract the issues caused by unbalanced prevalence (Supplementary Information S7 for further details). Finally, we removed the additional pseudo-absence locations and retained an equal subset of pseudo-absence locations as the spatially filtered presence locations to counteract problems arising from unbalanced prevalence^64^.

The landscape composition or LULC map of the Saurashtra region was originally obtained from Bhaskaracharya National Institute for Space Applications and Geo-informatics (BISAG-N), Gandhinagar, Gujarat. The original LULC map consisted of 18 classes for the whole Saurashtra region. We clipped the LULC to our Area of Interest (AOI) corresponding to the home rang polygons of the lesser floricans. We reclassified the LULC classes for our AOI into four major habitat types namely open scrub, dense scrub, grasslands, and croplands. We further undertook ground surveys to correctly assign each LULC class to the reclassified habitat types. In the next step, we further reclassified each habitat category on a continuous scale using the reclassify tool in ArcGIS 10.8.1 (Redlands, ESRI, California, USA). During the reclassification process, we assigned a value of 1 to the habitat category of interest and 0 to others to obtain a reclassified raster layer with raster values on a continuous scale. We used focal statistics and extracted values to the points within a 200-m radius of each lesser florican presence-absence point. Copernicus Night light pollution data was downloaded from (https://essd.copernicus.org/). There is a piece of growing evidence that night light interferes with bird navigation^65^, limits dispersal^66^, and may change the broad-scale distribution of birds^67^. All analyses pertaining to home ranges and statistical tests were performed using the R statistical programming language^56^ (http://www.r.project.org), and spatial data and temporal data, including daily movement and annual migration patterns, were analysed using Arc GIS 10.8.1(Redlands, ESRI, California, USA).

### Model fit and validation

We employed a k-fold partitioning approach to split the data into two sets: a training subset, comprising 80% of the locations, and a test subset, consisting of 20% of the locations. We shuffled the dataset randomly and split the data set into k = 10-folds. We randomly withheld 20% of the data (locations) as a test subset and the remaining as a training subset. We fitted a model on a training subset and evaluated it on the test subset to validate the predictions of the training model. To construct regression models, we used the training subset and specified the following equation:

$$ {\text{Logit}}\;P = {\text{ b}}_{0} = b_{1} x_{1} + b_{2} x_{2} + b_{3} x_{3} + \cdots b_{n} x_{n} = y, $$

where b_0_ = intercept of the regression model, b = coefficients of the independent variables, *x* = independent variables, and *y* = probability.

The minimum Akaike Information Criterion (AIC) was used for model selection^59,60^, and the final model was validated using a separate test data subset. Model accuracy was evaluated using misclassification error, model specificity, model sensitivity, and area under the curve (AUC) (see Supplementary Information S8 for details).

### Ethics declaration

All scientific research activities involved in this study were carried out after receiving approval from the competent authority (Ministry of Environment, Forests and Climate Change (MoEF&CC), Government of India (Letter No. F.No. 1-27/2020 WL dated 14th July 2020 and F.No. 1-27/2020 WL dated 10th September 2021). All experimental methods and works were carried out in accordance with relevant guidelines and regulations suggested by the Principal Chief Conservator of Forests (Wildlife) & Chief Wildlife Warden, Gujarat Forest Department, Government of Gujarat. Technical experts, experienced bird trappers, handlers, qualified and experienced veterinarians carried out the tagging work. Throughout the trapping and tagging process, every possible measure was implemented to mitigate disturbance and prioritise the well-being of the birds. Moreover, the study is reported in accordance with ‘Animal Research: Reporting of In Vivo Experiments’ (ARRIVE) guidelines as applicable.

The photographs in the Supplementary Information (SI) were taken and are owned by the Wildlife Division in Sasan-Gir. The corresponding author has the copyrights to these photographs and they are being shared for publication in an online open-access format as well as for offline printing. Permission was obtained to publish the images under open-access license of Creative Commons BY 4.0 DEED.

### Supplementary Information

Supplementary Information 1.

Supplementary Information 2.

Supplementary Information 3.

Supplementary Information 4.

Supplementary Information 5.

Supplementary Information 6.

Supplementary Information 7.

Supplementary Information 8.

## Data Availability

The datasets used or analysed in this study are available on reasonable request from the corresponding author.
