# Supplementary material for: Unravelling the secrets of lesser florican: a study of their home range and habitat use in Gujarat, India
Source: Sci Rep. 2023 Nov 4;13:19082. doi: 10.1038/s41598-023-46563-5 (PMC10625546; doi:10.1038/s41598-023-46563-5)
Supplement: Supplementary file 1 — Supplementary Information 1. [file 41598_2023_46563_MOESM1_ESM.docx]

**Supplementary Information S1: Partial plots of the habitat variables used to predict the occurrence of lesser floricans using generalized linear models.**


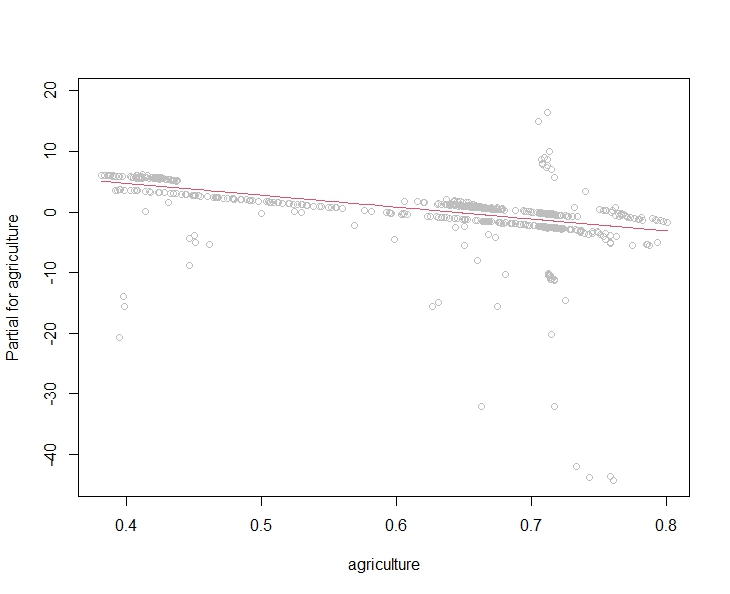


Fig.1: Partial dependence plot showing the marginal effect of agriculture on the predicted occurrence of lesser floricans


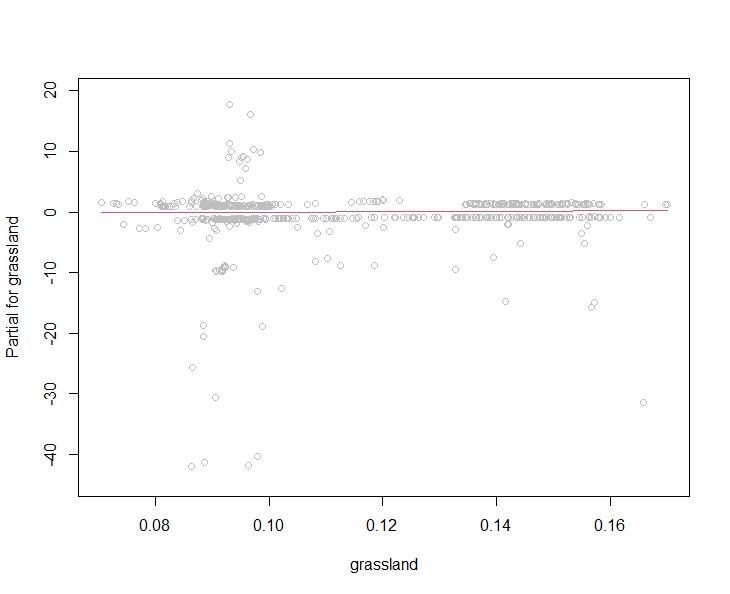


Fig. 2: Partial dependency plot showing the marginal effect of grassland habitat on the predicted occurrence of lesser floricans


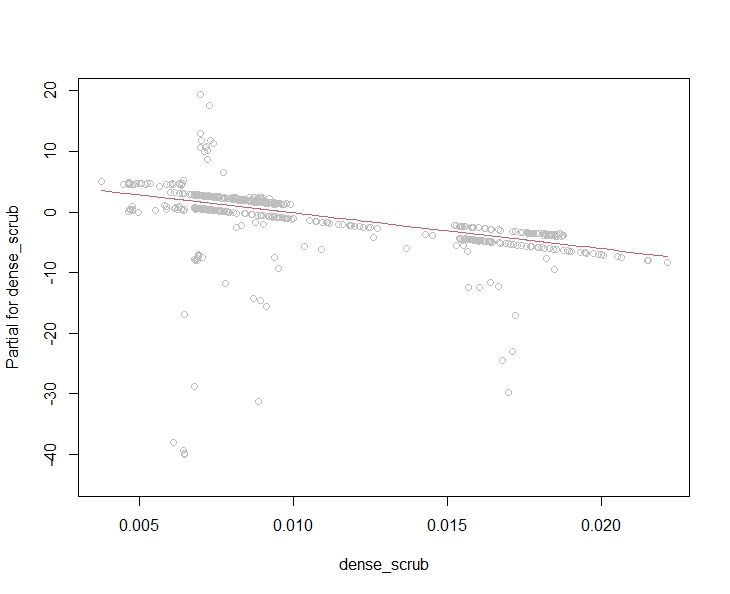


Fig. 3: Partial dependency plot showing the marginal effect of dense scrub habitat on the predicted occurrence of lesser floricans.


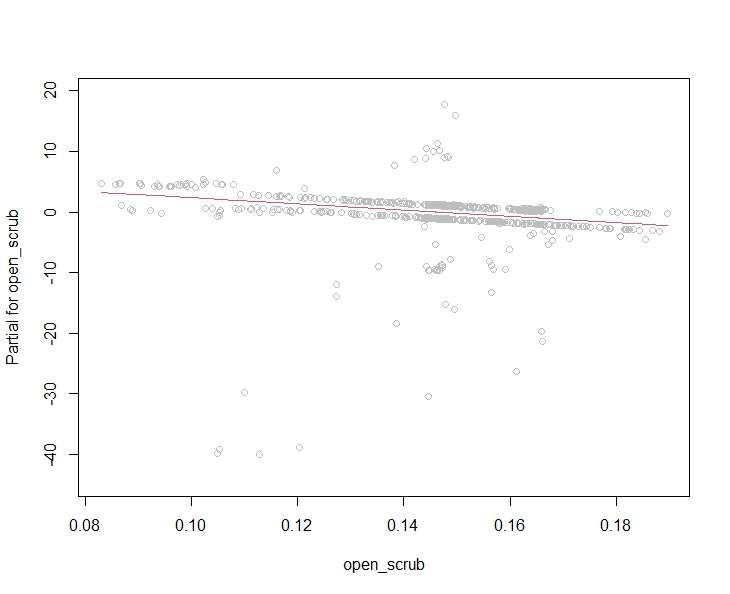


Fig. 4: Partial dependency plot showing the marginal effect of open scrub on the predicted occurrence of lesser floricans


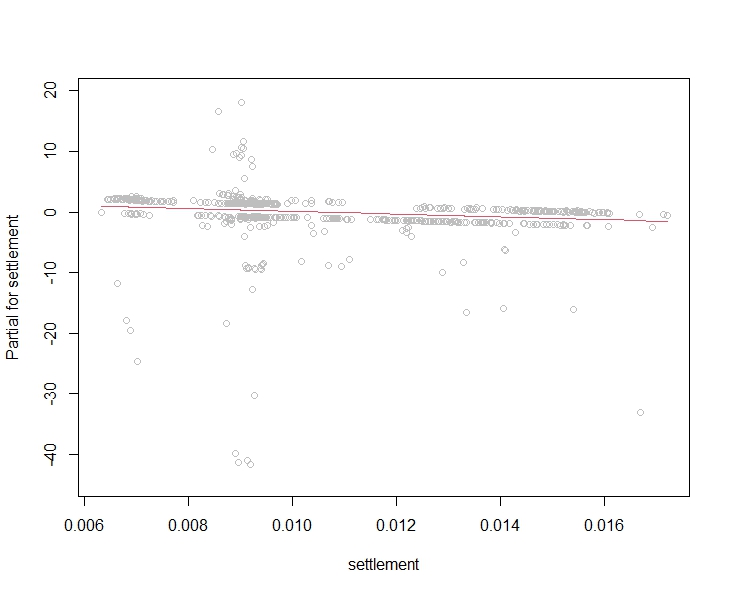


Fig. 5: Partial dependency plot showing the marginal effect of settlement on the predicted occurrence of lesser floricans


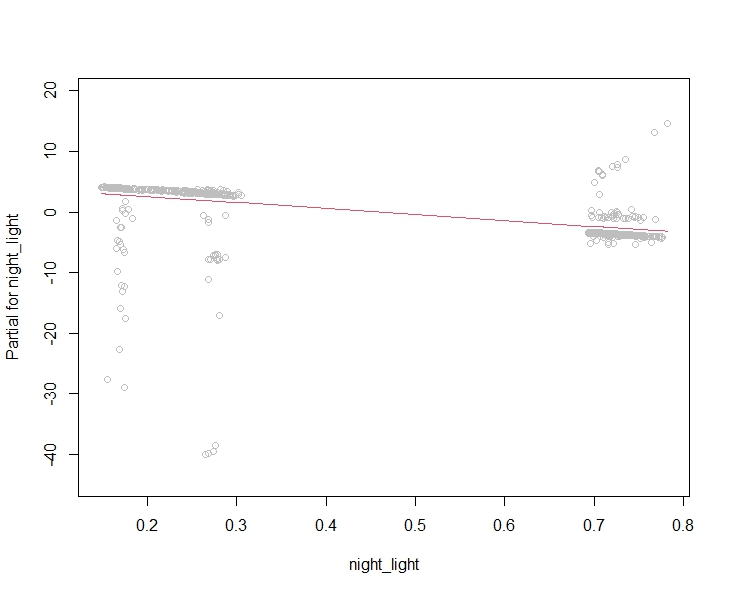


Fig. 6: Partial dependency plot showing the marginal effect of night light on the predicted occurrence of lesser floricans
