# Supplementary material for: Unravelling the secrets of lesser florican: a study of their home range and habitat use in Gujarat, India
Source: Sci Rep. 2023 Nov 4;13:19082. doi: 10.1038/s41598-023-46563-5 (PMC10625546; doi:10.1038/s41598-023-46563-5)
Supplement: Supplementary file 2 — Supplementary Information 2. [file 41598_2023_46563_MOESM2_ESM.docx]

**Supplementary Information S2: The administrative details of the stopovers used by LFM9, LFM10, and LFM3 during their migration.**

| **Sr. No.** | **Bird ID No.** | **State** | **District** | **Sub-district/Taluka** | **Village** | **Stopover days** | **Month-Year** |
| --- | --- | --- | --- | --- | --- | --- | --- |
| 1 | **LFM9** | Gujarat | Bhavnagar | Bhavnagar | Rajgadh | 1 | September 2022 |
| 2 |  | Gujarat | Bhavnagar | Vallabhipur | Moti Dharai | 34 | September-October 2022 |
| 3 |  | Gujarat | Bhavnagar | Vallabhipur | Italiya |  |  |
| 4 |  | Gujarat | Bhavnagar | Vallabhipur | Shahpur |  |  |
| 5 |  | Gujarat | Bhavnagar | Vallabhipur | Ratanpur |  |  |
| 6 |  | Gujarat | Bhavnagar | Umrala | Golrama | 1 | October 2022 |
| 7 |  | Gujarat | Bhavnagar | Sihor | Sedarda | 9 | October-November 2022 |
| 8 |  | Gujarat | Bhavnagar | Sihor | Kanad | 2 | November 2022 |
| 9 |  | Gujarat | Bhavnagar | Sihor | Sakhvadar | 1 |  |
| 10 |  | Gujarat | Bhavnagar | Sihor | Kajavadar |  |  |
| 11 |  | Gujarat | Bhavnagar | Sihor | Jambala | 1 |  |
| 12 |  | Gujarat | Bhavnagar | Talaja | Kundheli | 1 |  |
| 13 |  | Gujarat | Bhavnagar | Talaja | Bhungar | 4 |  |
| 14 |  | Gujarat | Bhavnagar | Talaja | Mangela |  |  |
| 15 |  | Gujarat | Bhavnagar | Talaja | Chopada | 2 |  |
| 16 |  | Gujarat | Valsad | Valsad | Gadaria | 1 |  |
| 17 |  | Maharashtra | Palghar | Vada | Gargaon | 9 |  |
| 18 |  | Maharashtra | Pune | Junnar | Khubi | 1 |  |
| 19 |  | Maharashtra | Pune | Junnar | Yedgaon | 1 |  |
| 20 |  | Maharashtra | Pune | Junnar | HivareTarf Narayangaon | 2 |  |
| 21 |  | Maharashtra | Pune | Khed | Jaulke Bk. | 1 |  |
| 22 |  | Maharashtra | Pune | Shirur | Karandi | 1 |  |
| 23 |  | Maharashtra | Pune | Haveli | Ashtapur | 1 |  |
| 24 |  | Maharashtra | Pune | Purandhar | Tekavadi | 1 |  |
| 25 |  | Maharashtra | Pune | Baramati | Murti | 1 |  |
| 26 |  | Maharashtra | Satara | Phaltan | Pimpalwadi | 1 |  |
| 27 |  | Maharashtra | Satara | Phaltan | Farandwadi | 2 |  |
| 28 |  | Maharashtra | Satara | Phaltan | Wathar (Nimbalkar) |  |  |
| 29 |  | Maharashtra | Satara | Phaltan | Girvi | 1 | December 2022 |
| 30 |  | Maharashtra | Satara | Man | Pangari | 2 |  |
| 31 |  | Maharashtra | Satara | Man | Virobanagar |  |  |
| 32 | **LFM10** | Gujarat | Ahmadabad | Barwala | Sodhi | 1 | October 2022 |
| 33 |  | Gujarat | Ahmadabad | Barwala | Navda | 5 |  |
| 34 |  | Gujarat | Bhavnagar | Bhavnagar | Velavadar | 26 | October-November 2022 |
| 35 |  | Gujarat | Bhavnagar | Bhavnagar | Adhelai | 1 | November 2022 |
| 36 |  | Gujarat | Bhavnagar | Sihor | Bhangadh | 1 |  |
| 37 |  | Gujarat | Bhavnagar | Bhavnagar | Bhadbhid | 1 |  |
| 38 |  | Gujarat | Bhavnagar | Vallabhipur | Mevasa | 7 |  |
| 39 |  | Gujarat | Bhavnagar | Bhavnagar | Sanes | 1 |  |
| 40 |  | Gujarat | Surat | Olpad | Bhadol | 1 |  |
| 41 |  | Gujarat | Surat | Vyara | Pithadara | 1 |  |
| 42 |  | Gujarat | The Dangs | The Dangs | Dhulchond | 1 |  |
| 43 |  | Gujarat | The Dangs | The Dangs | Payarpada | 1 |  |
| 44 |  | Maharashtra | Nasik | Kalwan | Sarale Digar | 1 |  |
| 45 |  | Maharashtra | Nasik | Kalwan | Kurdane (Otur) | 1 |  |
| 46 |  | Maharashtra | Nasik | Chandvad | Vadner Bhairao | 1 |  |
| 47 |  | Maharashtra | Nasik | Niphad | Karanjgaon | 1 |  |
| 48 |  | Maharashtra | Nasik | Sinnar | Deopur | 2 |  |
| 49 |  | Maharashtra | Ahmadnagar | Sangamner | Wadzari Kh. | 1 |  |
| 50 |  | Maharashtra | Ahmadnagar | Rahuri | Varshinde | 1 |  |
| 51 |  | Maharashtra | Ahmadnagar | Nagar | Nimgaon Ghana | 1 |  |
| 52 |  | Maharashtra | Ahmadnagar | Nagar | Akolner | 1 |  |
| 53 |  | Maharashtra | Ahmadnagar | Shrigonda | Kothul | 1 |  |
| 54 |  | Maharashtra | Ahmadnagar | Karjat | Rakshaswadi Kh. | 1 |  |
| 55 |  | Maharashtra | Solapur | Karmala | Kumbhargaon | 5 | November-December 2022 |
| 56 | **LFM3** | Gujarat | Kutch | Abdasa | Aida | 2 | June 2022 |
| 57 |  | Gujarat | Kutch | Abdasa | Jagaliya | 1 |  |
| 58 |  | Gujarat | Kutch | Lakhpat | Dedrani | 1 |  |
| 59 |  | Gujarat | Kutch |  | Greater Rann of Kutch | 14 | July 2022 |
