# Supplementary material for: Unravelling the secrets of lesser florican: a study of their home range and habitat use in Gujarat, India
Source: Sci Rep. 2023 Nov 4;13:19082. doi: 10.1038/s41598-023-46563-5 (PMC10625546; doi:10.1038/s41598-023-46563-5)
Supplement: Supplementary file 3 — Supplementary Information 3. [file 41598_2023_46563_MOESM3_ESM.docx]

| **ID** | **Cropland** | **Open Scrub** | **Dense Scrub** | **Grassland** |
| --- | --- | --- | --- | --- |
| LFM1 | 22.19 | 3.61 | - | - |
| LFM2 | 30.90 | - | - | 68.98 |
| LFM3 | 48.59 | 1.77 | 14.50 | 31.41 |
| LFM4 | 25.65 | - | - | 74.34 |
| LFM5 | 35.77 | 5.79 | 0.55 | 52.50 |
| LFM7 | - | - | - | 100 |
| LFM8 | 62.12 | 0.53 | - | 35.46 |
| LFM9 | 66.44 | 5.28 | - | 26.94 |
| LFM10 | 51.11 | 34.70 | - | 10.26 |
| LFM11 | 78.11 | 16.50 | 2.06 | - |

**Supplementary Information S3:Percentage of different habitat types within the home range polygons of each lesser florican.**

- denotes ‘not present’
