# Supplementary material for: Unravelling the secrets of lesser florican: a study of their home range and habitat use in Gujarat, India
Source: Sci Rep. 2023 Nov 4;13:19082. doi: 10.1038/s41598-023-46563-5 (PMC10625546; doi:10.1038/s41598-023-46563-5)
Supplement: Supplementary file 4 — Supplementary Information 4. [file 41598_2023_46563_MOESM4_ESM.docx]

**Supplementary Information S4: The tagging details of the lesser floricans in Blackbuck National Park (BNP), Velavadar, Bhavnagar District and Kutch Bustard Sanctuary (KBS), Abdasa, Kutch, Gujarat, India.**

| **Species ID** | **Date of Tagging** | **Tag No.** | **Time of Tagging (IST)**  **Hrs** | **Time of Tagging (UTC)**  **Hrs** | **Place of Tagging** | **Type of Tag** | **Weight of Tag**  **(in grams)** | **Weight of Bird (in grams)** | **Remarks (if any)** |
| --- | --- | --- | --- | --- | --- | --- | --- | --- | --- |
| LFM1 | 04/09/2020 | 206474 | 07:00 | 01:30 | BNP, Velavadar | Solar Doppler PTT (Argos Satellite Tag) | 12 | 506 | The tag showed steady activity values since 27^th^ Feb, 2022 to 1^st^ March, 2022. This indicates either mortality of the bird or detachment of the tag. |
| LFM2 | 16/09/2021 | 213385 | 08:00 | 02:30 | KBS, Abdasa | Ornitella GSM Tag | 10 | 440 | It showed restricted movement on 27/10/2021. On ground verification, it was found entangled in a net placed around the farm; it was rescued and treated but unfortunately died on 28/10/2021. |
| LFM3 | 17/09/2021 | 213386 | 11:00 | 05:30 | KBS, Abdasa | Ornitella GSM Tag | 10 | 498 | The bird showed restricted movement on 11^th^ Feb, 2022. Local field staff was informed about the same. The team on reaching found that the bird was entangled in the fishing net surrounding the farm. The bird was rescued and examined thoroughly. Fortunalety there were no injury and hence released back. |
| LFM4 | 25/09/2021 | 213387 | 09:00 | 03:30 | KBS, Abdasa | Ornitella GSM Tag | 10 | 450 | Suddenly showed error on 26/12/2021 and no communication since then. |
| LFM5 | 03/10/2021 | 206473 | 07:10 | 01:40 | KBS, Abdasa | Solar Doppler PTT (Argos Satellite Tag) | 12 | 470 | - |
| LFM6 | 17/08/2022 | 222487 | 11:30 | 05:00 | BNP, Velavadar | Ornitella GSM Tag | 10 | 462 | - |
| LFM7 | 17/08/2022 | 222488 | 11:47 | 05:17 | BNP, Velavadar | Ornitella GSM Tag | 10 | 473 | - |
| LFM8 | 20/08/2022 | 222486 | 02:59 | 08:29 | BNP, Velavadar | Ornitella GSM Tag | 10 | 499 | - |
| LFM9 | 21/08/2022 | 222484 | 09:04 | 02:34 | BNP, Velavadar | Ornitella GSM Tag | 10 | 450 | - |
| LFM10 | 21/08/2022 | 222485 | 09:12 | 02:42 | BNP, Velavadar | Ornitella GSM Tag | 10 | 466 | - |
| LFM11 | 25/08/2022 | 213385 | 07:24 | 12:54 | BNP, Velavadar | Ornitella GSM Tag | 10 | 459 | - |
