# Supplementary material for: Unravelling the secrets of lesser florican: a study of their home range and habitat use in Gujarat, India
Source: Sci Rep. 2023 Nov 4;13:19082. doi: 10.1038/s41598-023-46563-5 (PMC10625546; doi:10.1038/s41598-023-46563-5)

LFM2 got entangled in a fishnet laid by the farmer around his agriculture field for crop protection (Photos taken during the rescue work). Photos by Wildlife Division, Sasan-Gir.


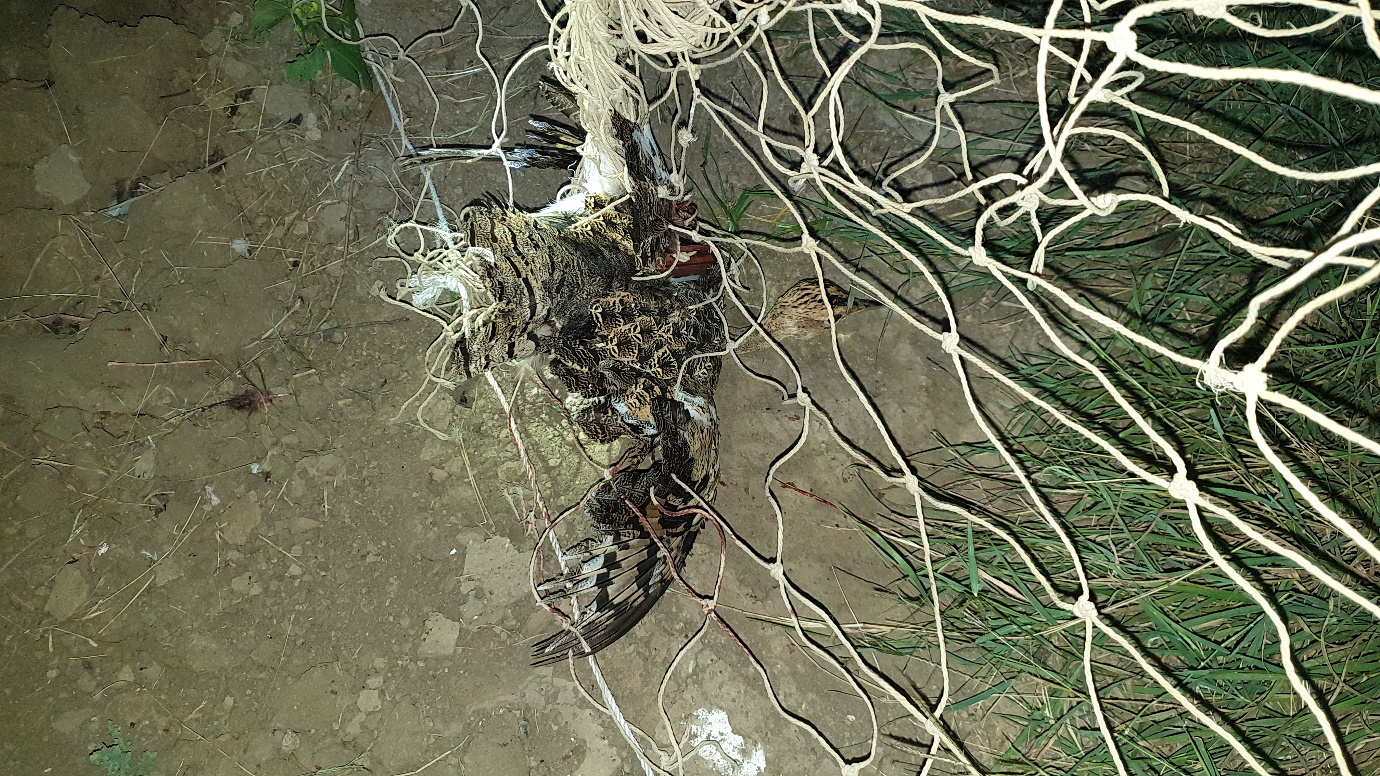


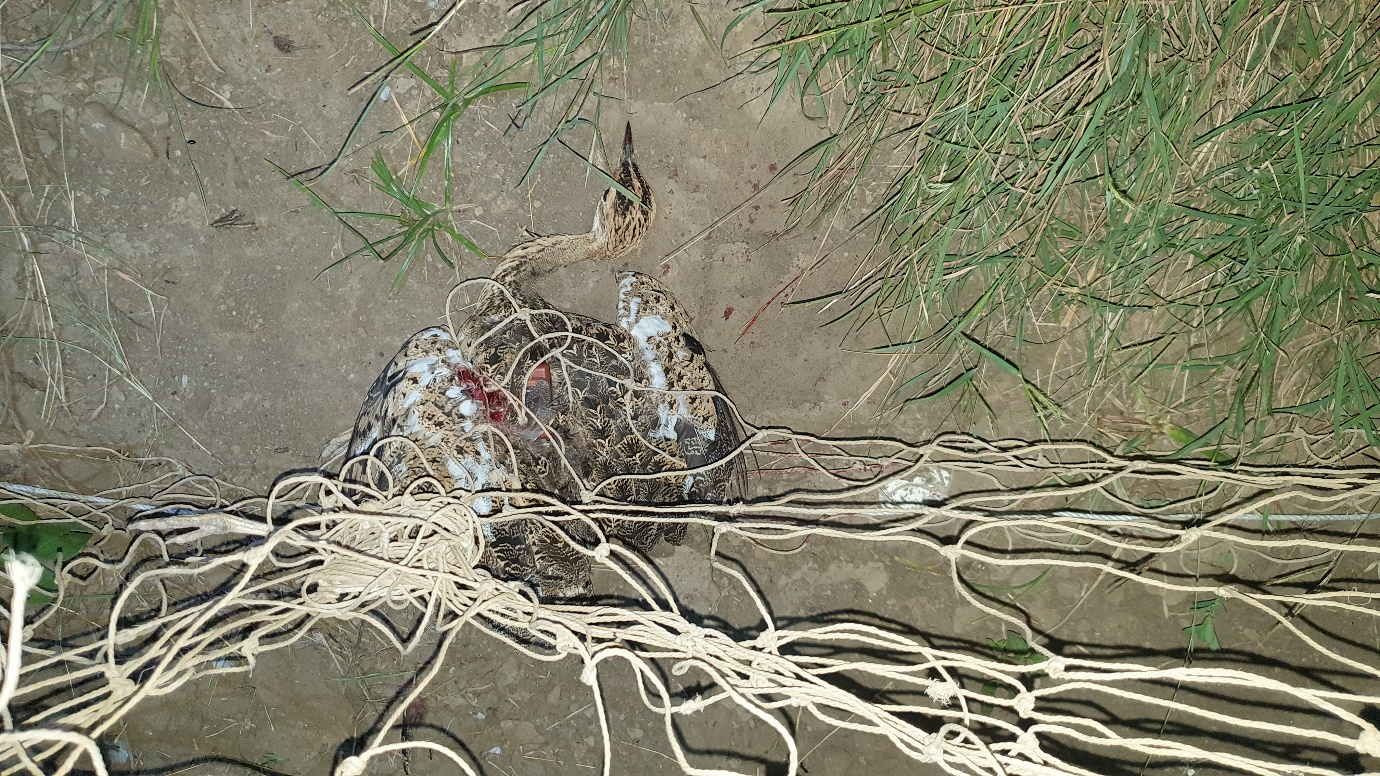

Supplement: Supplementary file 5 — Supplementary Information 5. [file 41598_2023_46563_MOESM5_ESM.docx]
