# Supplementary material for: Unravelling the secrets of lesser florican: a study of their home range and habitat use in Gujarat, India
Source: Sci Rep. 2023 Nov 4;13:19082. doi: 10.1038/s41598-023-46563-5 (PMC10625546; doi:10.1038/s41598-023-46563-5)
Supplement: Supplementary file 6 — Supplementary Information 6. [file 41598_2023_46563_MOESM6_ESM.docx]

**Supplementary Information S6: Photographic details of the complete tagging process. Photos by Wildlife Division, Sasan - Gir.**


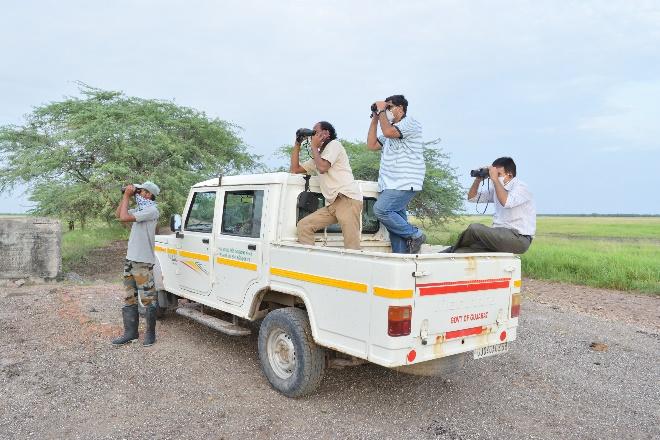

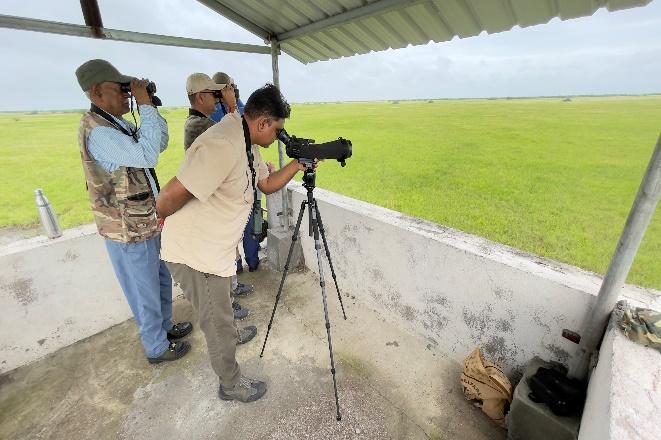


**Photo 1: The tagging team members observing the Lesser Floricans at display sites.**


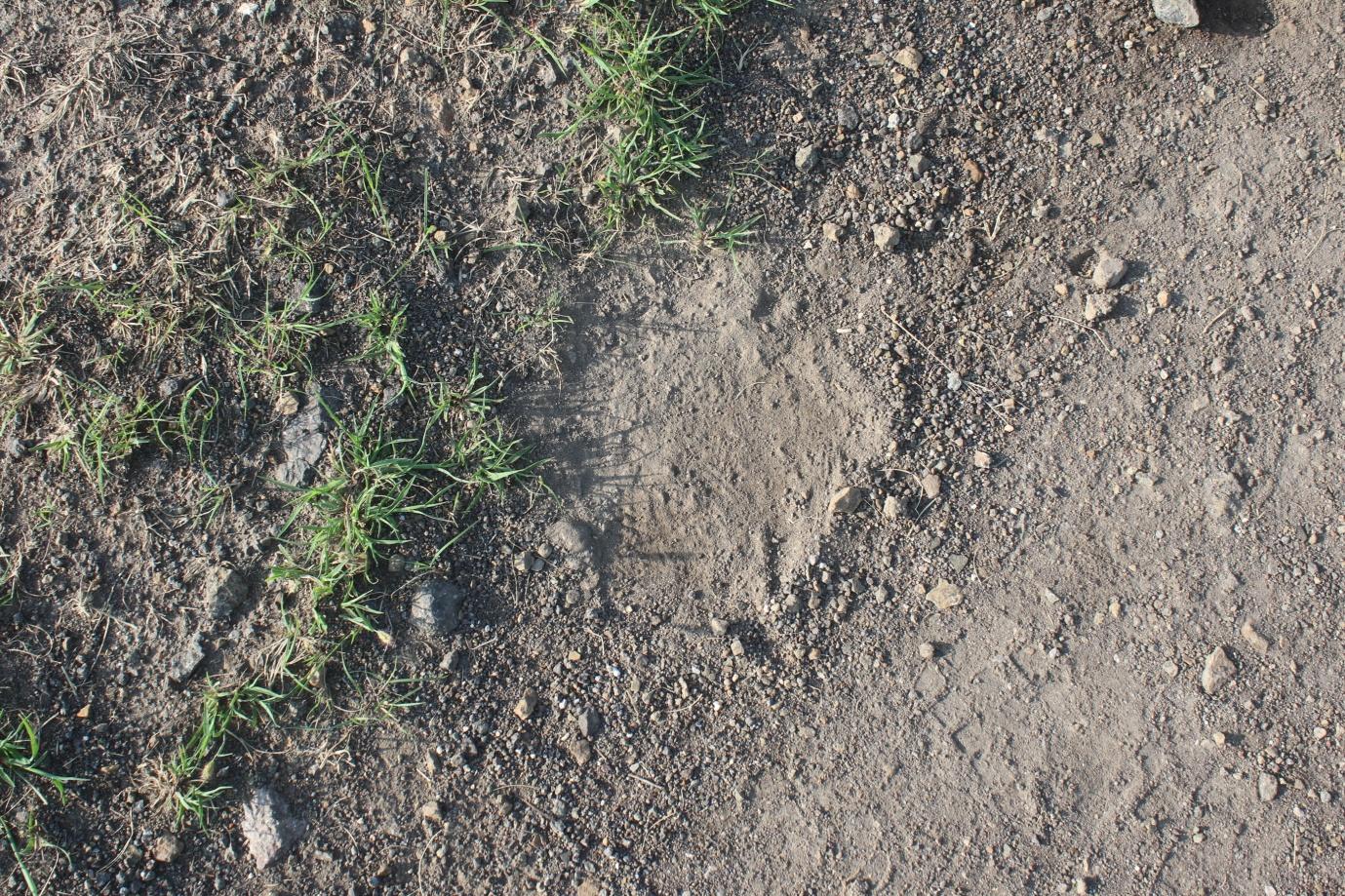


**Photo 2: A typical ground cover at the display site of the male Lesser Florican.**

**
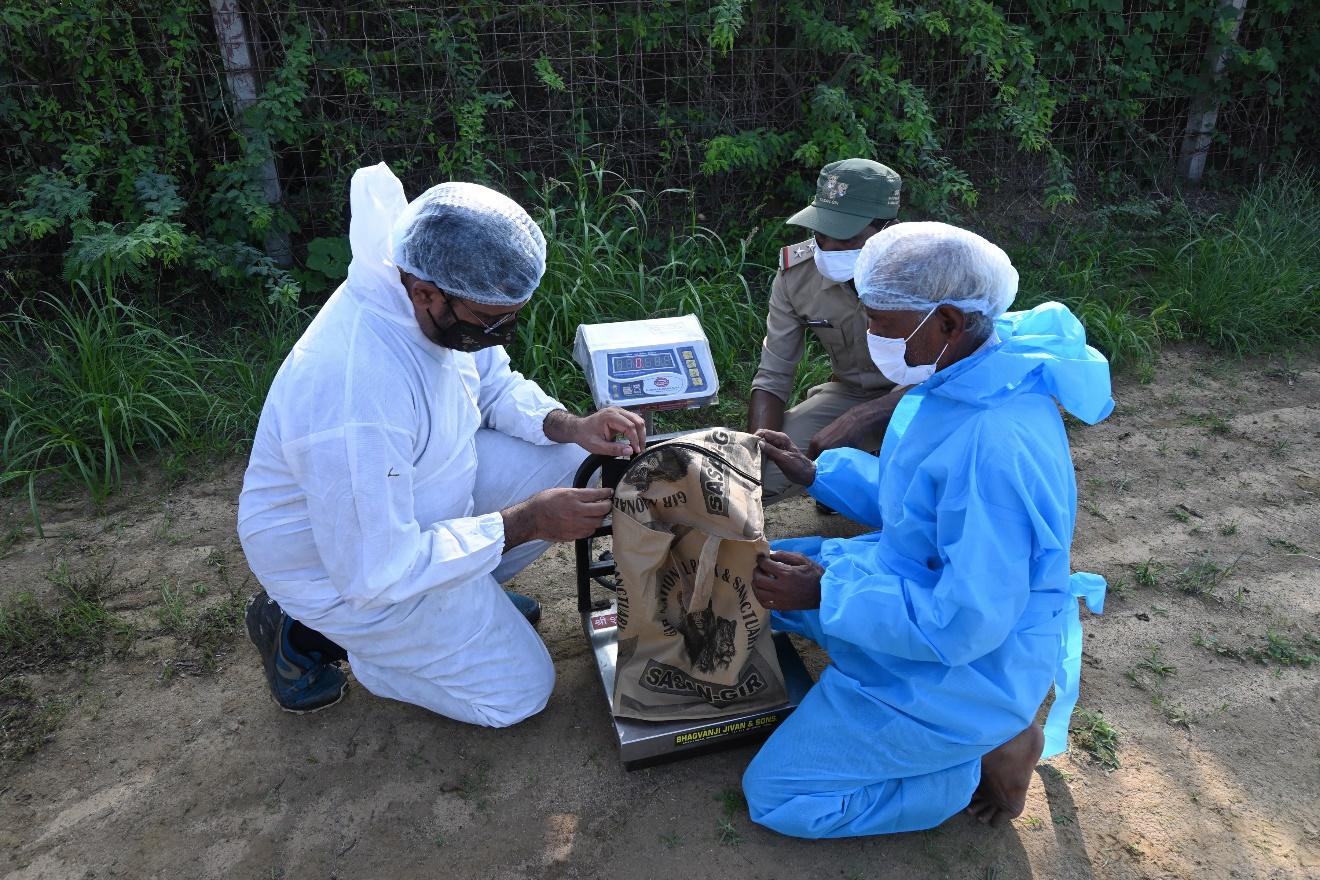
**

**Photo 3: The captured bird was kept in the bird bag to keep it calm and safe. Each bird was weighed before the deployment of the transmitter.**

**
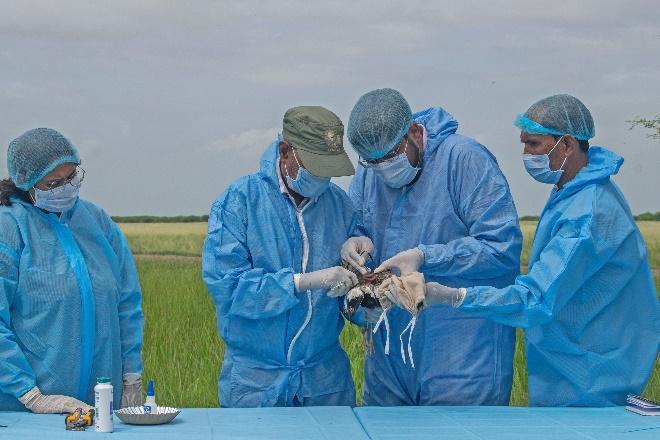

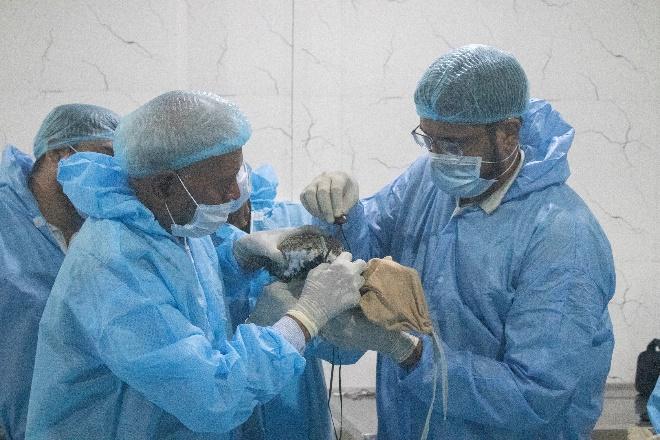
**

**Photo 4: During the tagging process, if needed, a specially designed head cap was used to cover the bird’s head to keep it calm.**

**
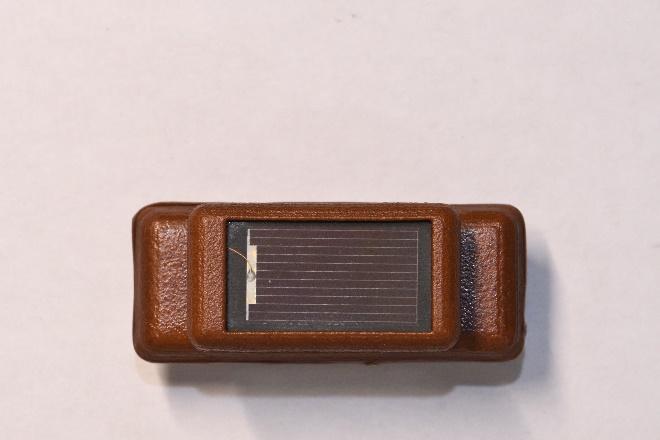

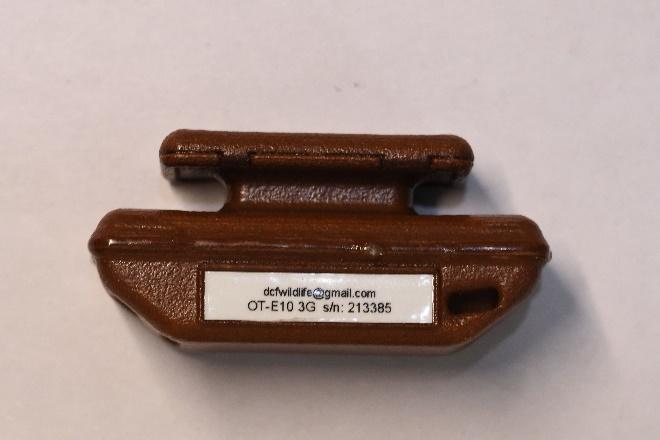
**

**Photo 5: Dorsal and lateral view of the GPS-GSM transmitter deployed on Lesser Floricans on the back using a Teflon harness.**


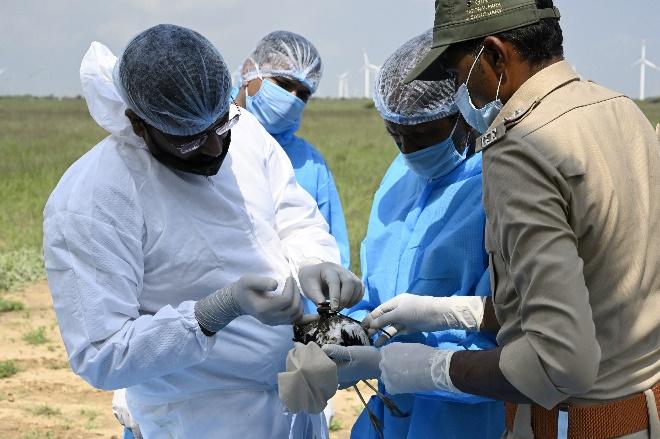

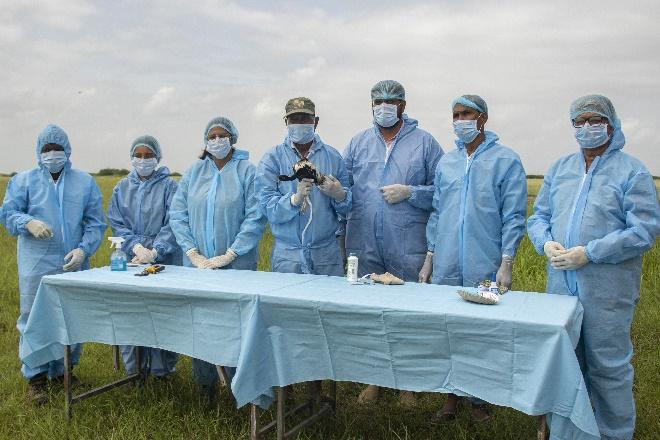


**Photo 6: On-field tagging work under the supervision of senior forest officials**


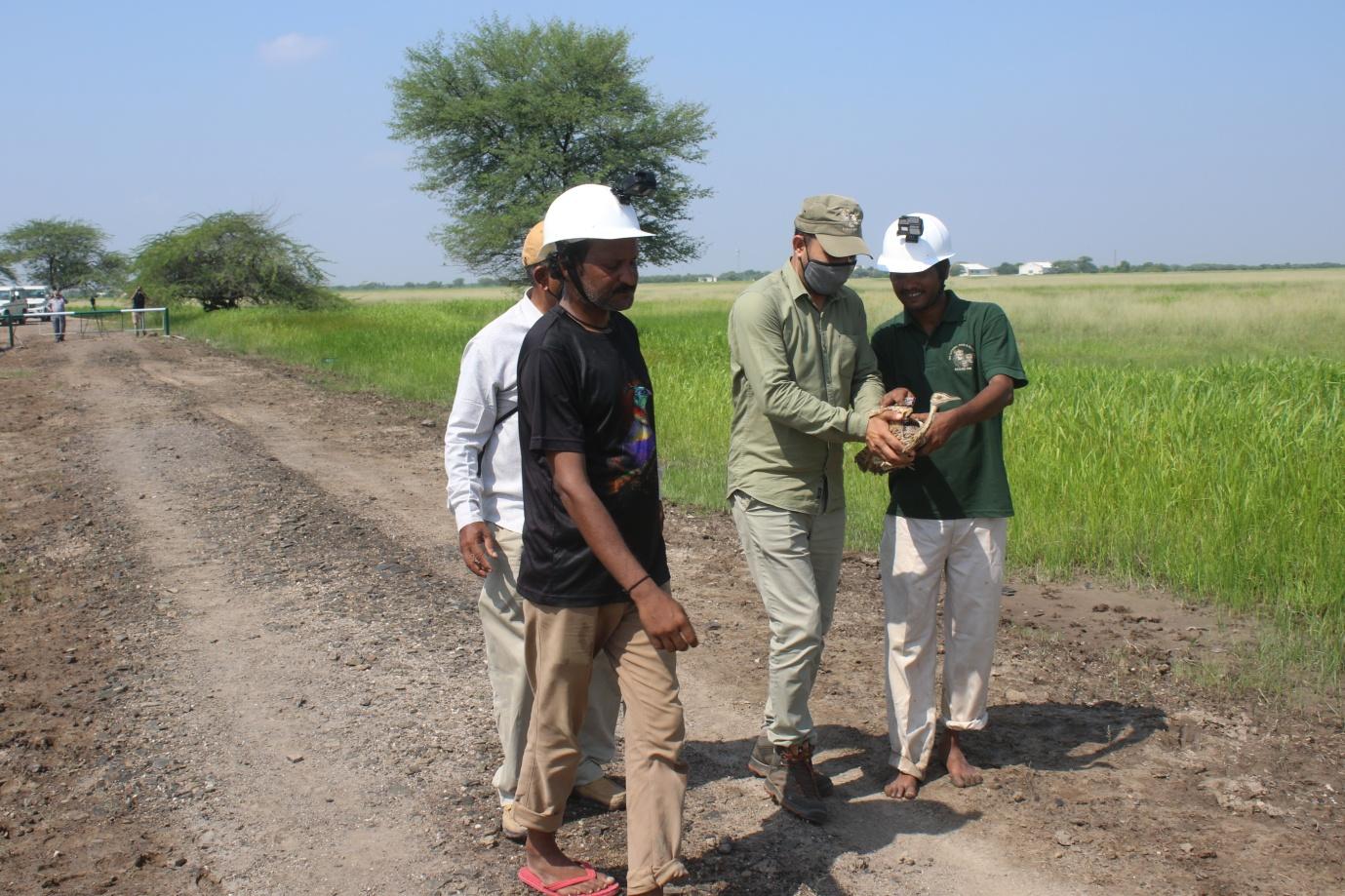

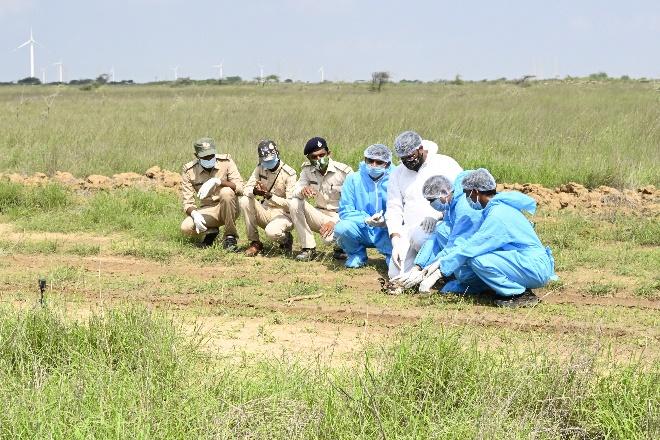

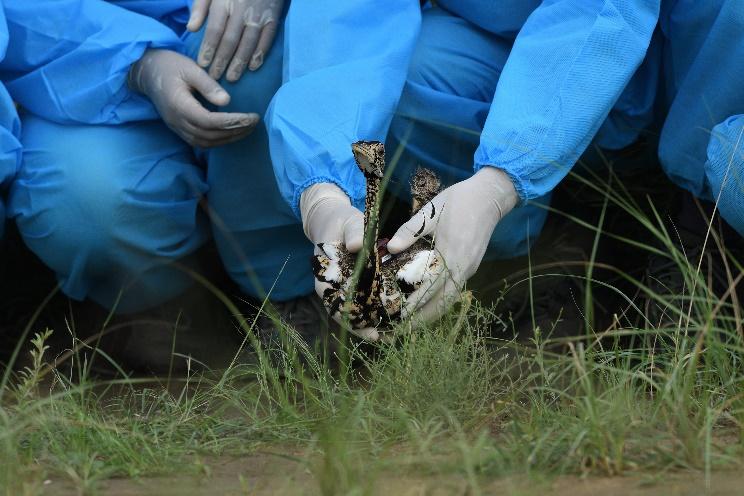

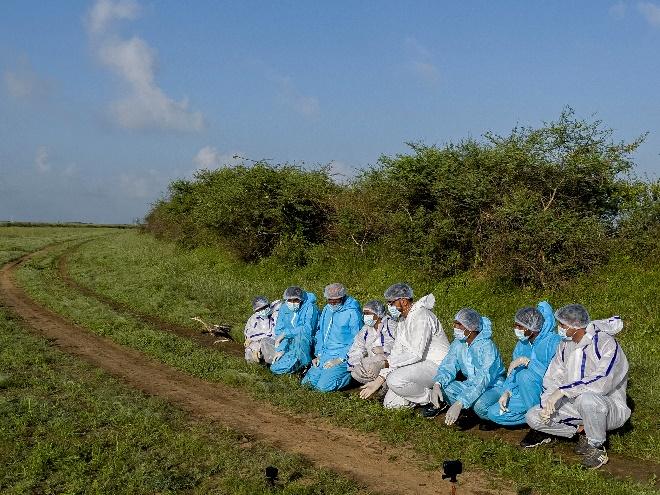


**Photo 7: The Lesser Floricans being released after tagging.**


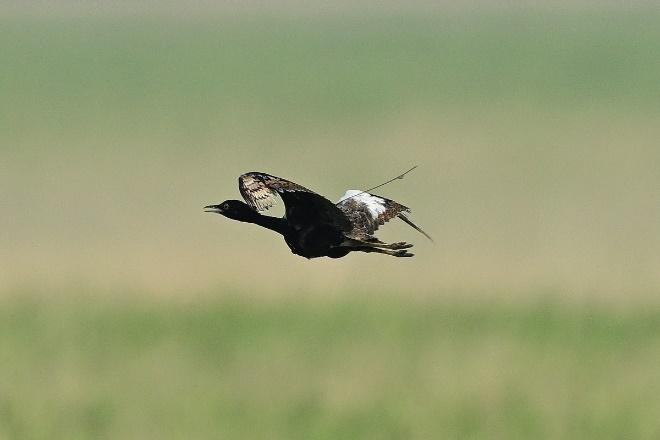

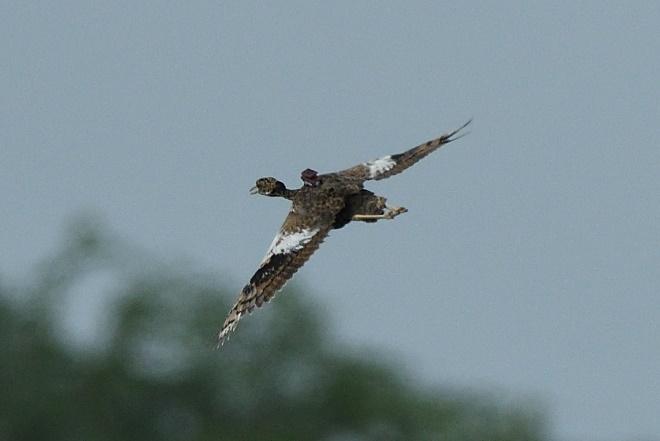

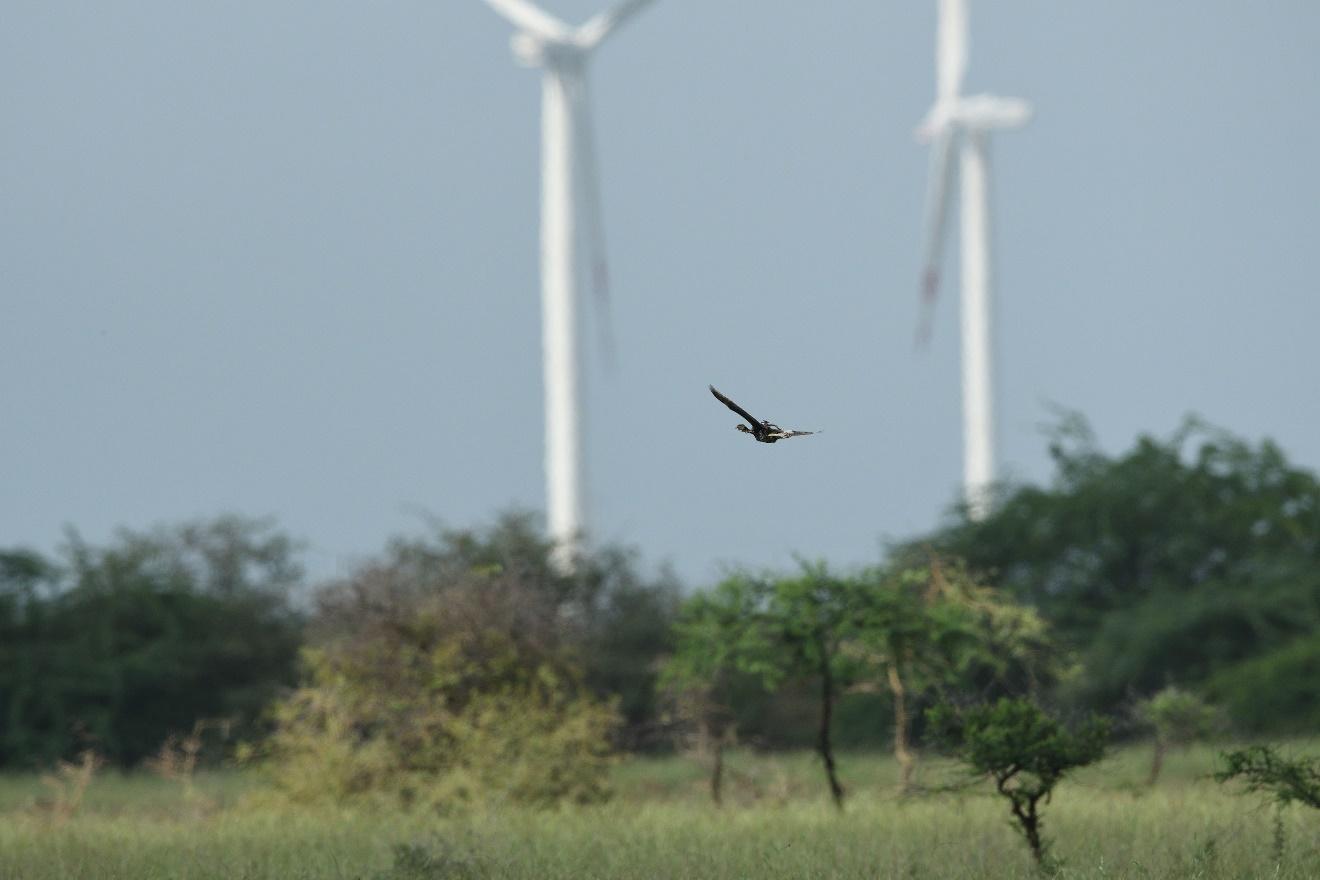


**Photo 8: Successful release of the tagged Lesser Floricans.**

**
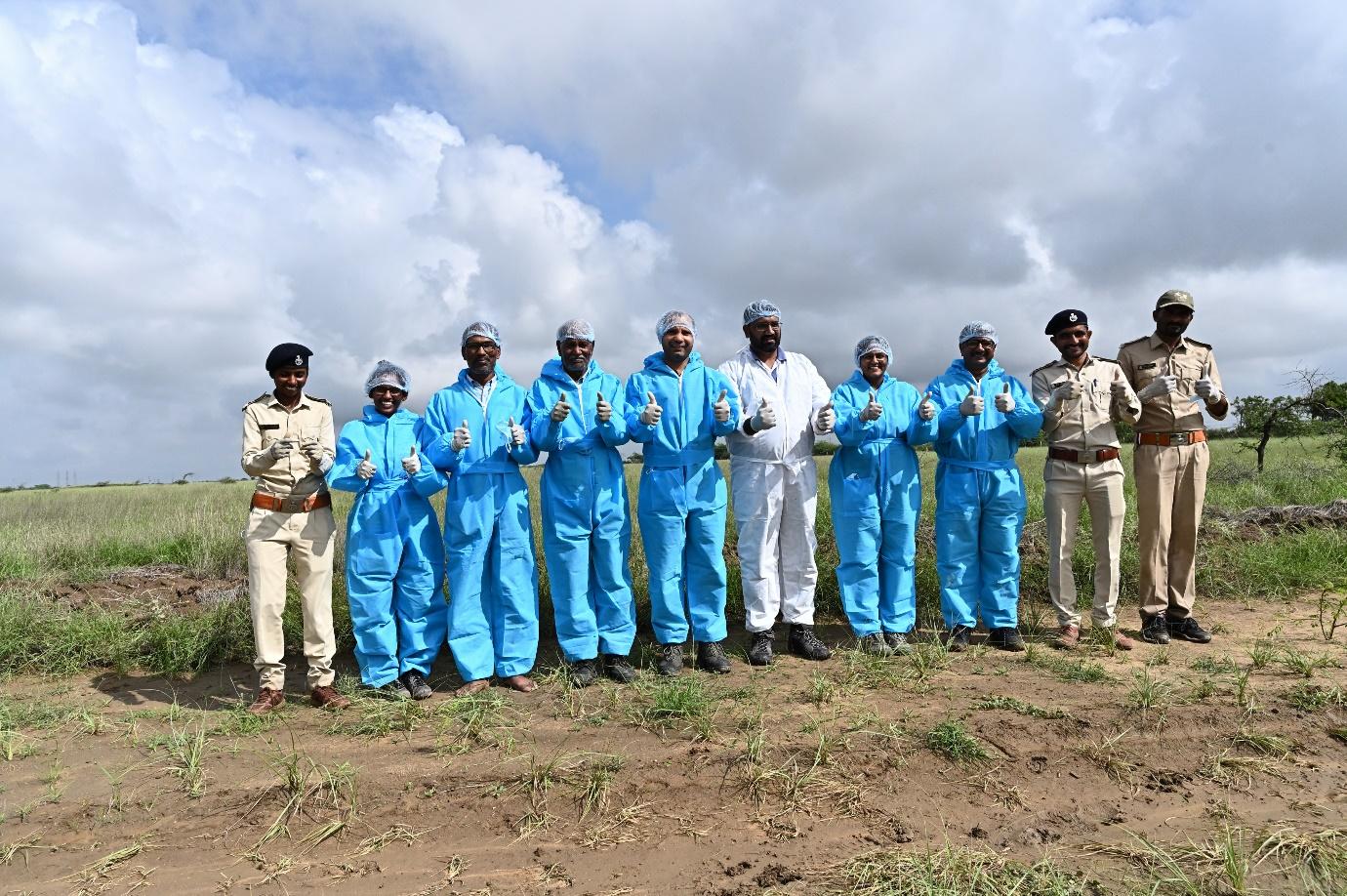
**

**
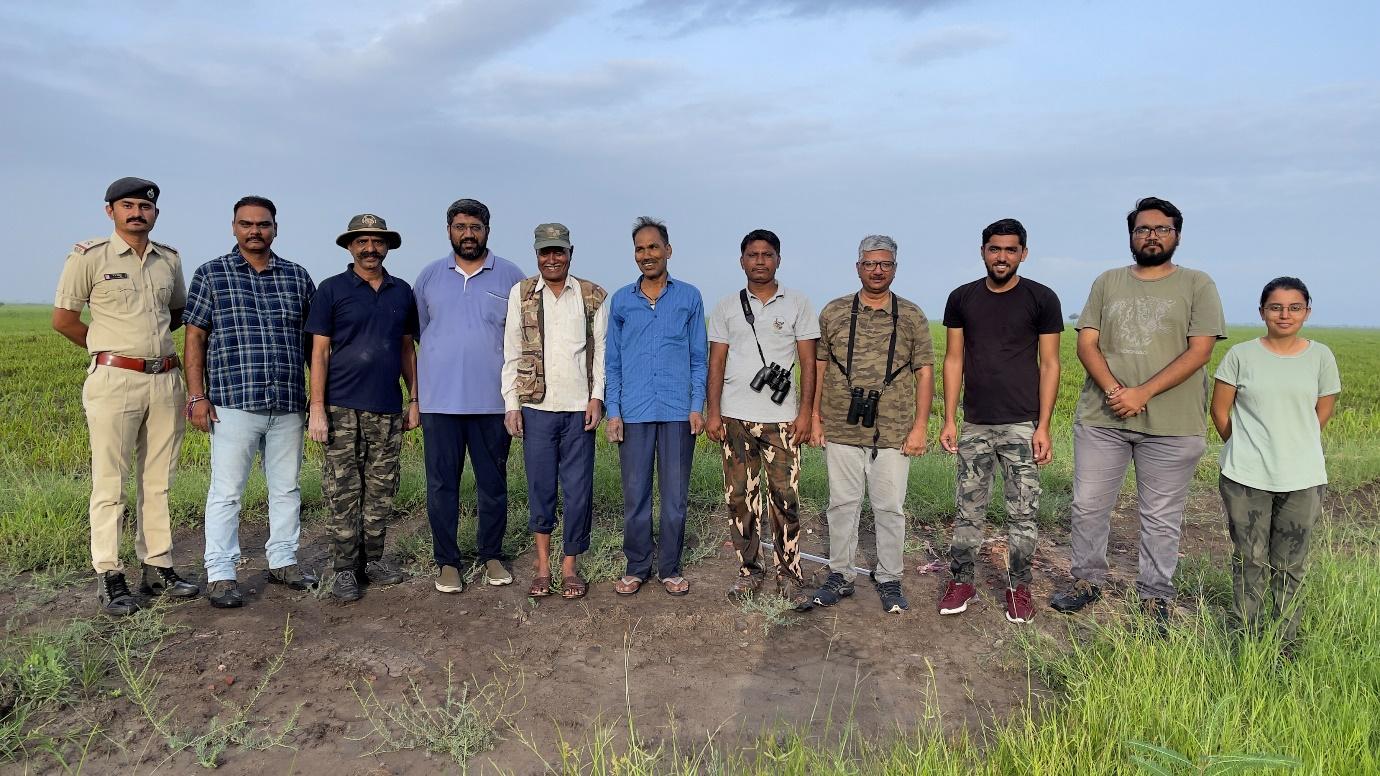
**

**Photo 9: Happy teams after the successful release of the birds.**
