# Supplementary material for: Unravelling the secrets of lesser florican: a study of their home range and habitat use in Gujarat, India
Source: Sci Rep. 2023 Nov 4;13:19082. doi: 10.1038/s41598-023-46563-5 (PMC10625546; doi:10.1038/s41598-023-46563-5)

**Supplementary Information S7: The spatial autocorrelation report as derived from the Spatial Autocorrelation tool in ArcGIS (version 10.8.1)**

The spatial data is associated with the inherent bias commonly referred to as spatial autocorrelation (Dormann et al. 2007). The phenomenon of spatial autocorrelation occurs due to the non-independency of variables sampled at nearby locations from each other (Tobler 1970). We used two methods to correct for the spatial autocorrelation in the Lesser florican presence locations. At first, we implemented spatial filtering using the SDM toolbox (Brown 2014) in ArcGIS to reduce the spatial bias in presence records whereby satellite telemetry records were spatially rarefied at 200 meters from each other. Secondly, we tested whether or not the spatially rarefied occurrence records assumed the random distribution after implementing spatial filtering by calculating Global Moran’s I (Moran 1950) using Spatial Autocorrelation tool in ArcGIS.


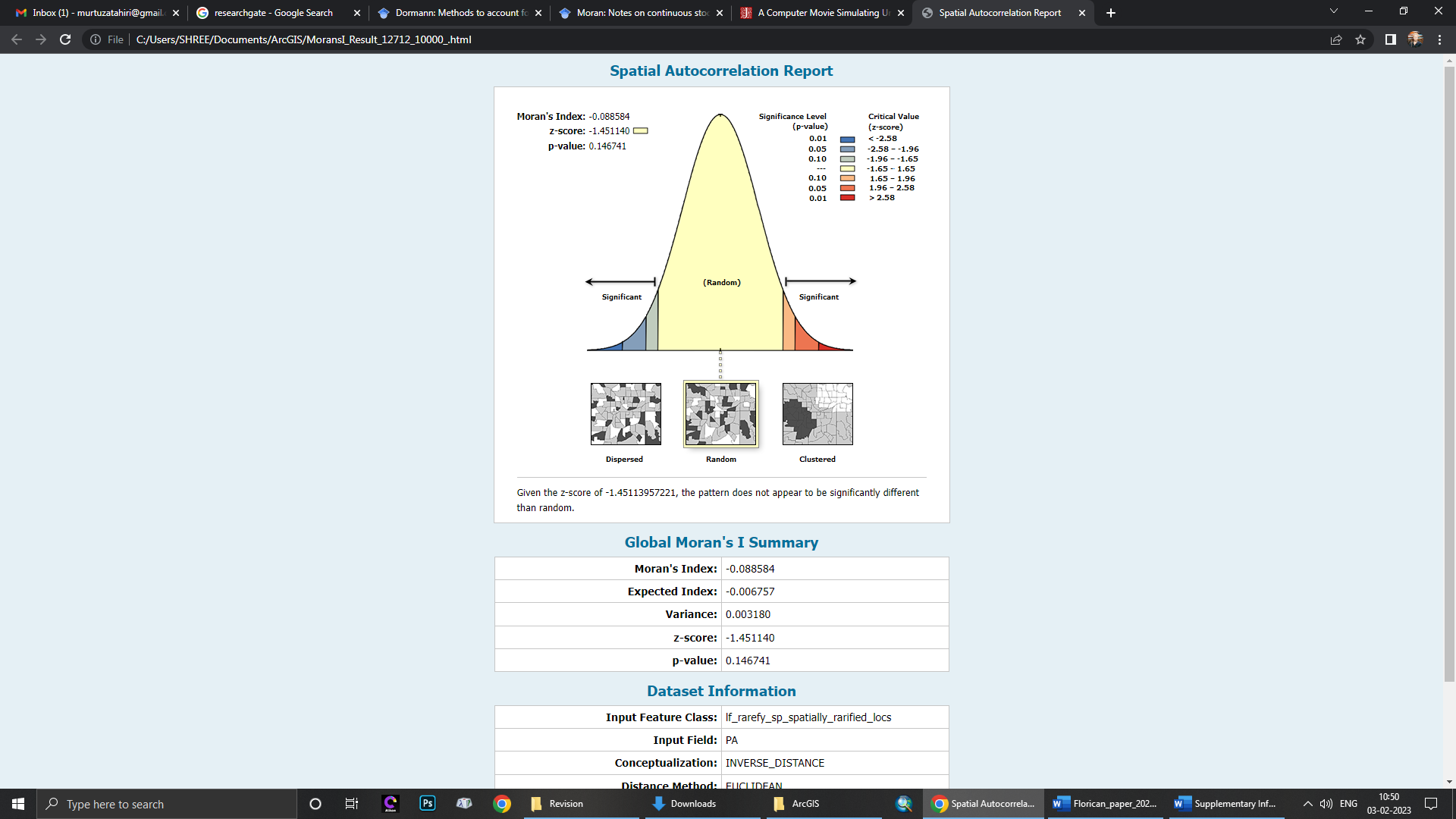

Supplement: Supplementary file 7 — Supplementary Information 7. [file 41598_2023_46563_MOESM7_ESM.docx]
