# Supplementary material for: Unravelling the secrets of lesser florican: a study of their home range and habitat use in Gujarat, India
Source: Sci Rep. 2023 Nov 4;13:19082. doi: 10.1038/s41598-023-46563-5 (PMC10625546; doi:10.1038/s41598-023-46563-5)
Supplement: Supplementary file 8 — Supplementary Information 8. [file 41598_2023_46563_MOESM8_ESM.docx]

**Supplementary Information S8: Model validation matrix of the fine-scale habitat selection model (GLM) of Lesser Florican.**

| **Performance matrix** | **value** |
| --- | --- |
| Area under curve (AUC) | 0.92 |
| Specificity | 0.84 |
| Sensitivity | 0.93 |
| Mis-classification error | 0.10 |
| Accuracy | 0.89 |
| P-value | 0.001 |


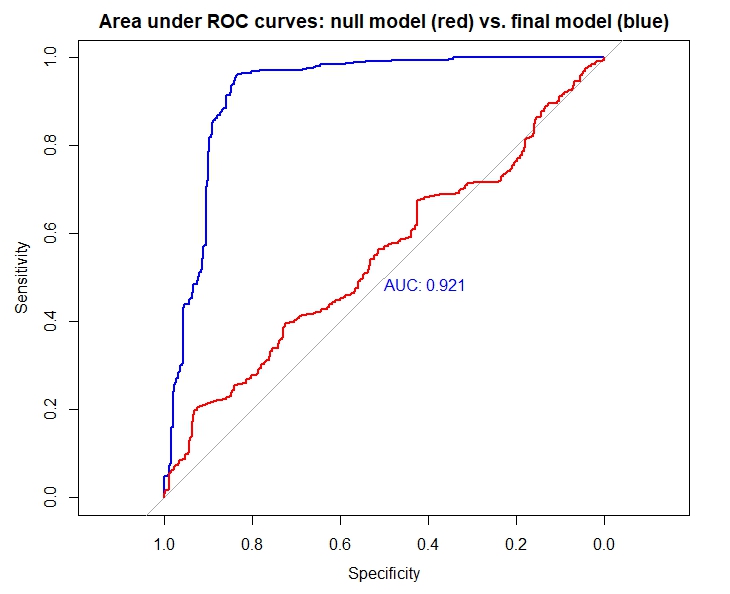


Fig. 1: Area under ROC Curve (AUC) for the final model (blue colour) against the null model (red colour).
